# Ferroptosis–neuroinflammation interplay: mechanistic pathways and therapeutic opportunities in central nervous system disorders

**DOI:** 10.3389/fnagi.2026.1874307

**Published:** 2026-09-03

**Authors:** Khuzin Dinislam, Valeriy A. Kataev, Mohammad Rehan Ajmal, Ayman S. Amer, Pratibha Prasad, Azna Zuberi

**Affiliations:** 1 Department of General Chemistry, Bashkir State Medical University, Republic of Bashkortostan, Ufa, Russia; 2 Bashkir State Medical University, Ministry of Health of the Russian Federation, Ufa, Russia; 3 Ufa University of Science and Technology, Ufa, Russia; 4 Physical Biochemistry Research Laboratory, Department of Biochemistry, Faculty of Science, University of Tabuk, Tabuk, Saudi Arabia; 5 Department of Basic Medical and Dental Sciences, College of Dentistry, Ajman University, Ajman, United Arab Emirates; 6 Department of Obstetrics and Gynecology, Northwestern University, Chicago, IL, United States

**Keywords:** aging and longevity, ferroptosis, healthspan, neuroinflammation, nutritional intervention, precision nutrition

## Abstract

Oxidative stress, iron dyshomeostasis, and chronic neuroinflammation are the principal mechanisms involved in the progression of central nervous system (CNS) disorders, which are a significant source of death and chronic disability worldwide. This review focuses on understanding the mechanistic relationship between ferroptosis and neuroinflammation in central nervous system (CNS) disorders, which involves the molecular mechanisms, their role in the pathogenesis of the disease, and therapeutic opportunities available. Ferroptosis is a regulated form of cell death triggered by iron-catalyzed lipid peroxidation, glutathione depletion and inactivation of glutathione peroxidase 4 (GPX4), resulting in neuronal injury. The increased generation of ROS in the cell due to the presence of excess iron catalyzes Fenton chemistry, which leads to lipid peroxidation of the cell membrane and ferroptotic cell death, and damage-associated molecular patterns originating from the ferroptotic cell death promote the activation of NF-κB-dependent inflammatory signaling pathways and inflammasome formation. Excessive dysregulations involving the hepcidin-ferroportin axis, DMT1–transferrin transport, ferritin–NCOA4-mediated ferritinophagy, and regulatory pathways like Nrf2, IRP/IRE, and SIRT1 intensify oxidative stress, ferroptosis and neuroinflammation in neurodegenerative and acute CNS disorders. The role of several key molecular mediators such as SIRT1, Nrf2, NF-κB, iNOS/NO●, and COX-2 in connecting ferroptotic and inflammatory signaling pathways is also discussed in this review. This understanding of the mechanism of these players gives a mechanistic framework for the development of targeted therapeutic and nutritional interventions to reduce neuronal damage and help slow the progression of CNS disorders.

## 1 Introduction

Iron plays a crucial role in several biological functions, such as the transport of oxygen, the synthesis of DNA, the production of neurotransmitters, and the metabolism of several enzymes (Skalnaya and Skalny, 2018; Islam et al., 2023). In the central nervous system (CNS), iron has essential functions during neuronal maturation, synaptic plasticity, myelination, and mitochondrial energy metabolism (Hidalgo et al., 2007; Muñoz and Humeres, 2012). Iron is, however, easily available to change its oxidation state, and excess iron in the cell will easily generate toxic reactive oxygen species (ROS) via the Fenton and Haber–Weiss reactions (Kehrer, 2000; Das et al., 2015). Iron overload-induced oxidative stress is believed to be a significant factor in neuronal dysfunction and cell death in many neurodegenerative disorders (Bains and Shaw, 1997). This disruption of delicate iron balance can therefore cause iron overload, which might trigger ferroptosis, an iron-dependent and non-apoptotic mode of regulated cell death, where lipid peroxidation is excessive and causes membrane damage (Latunde-Dada, 2017). Ferroptosis is mainly driven by iron-induced oxidative damage of membrane PUFAs, which results in unique molecular signatures and regulatory checkpoints compared to other forms of cell death such as apoptosis, necrosis, and autophagy (Su et al., 2019; Chen et al., 2021). The Global Burden of Disease (GBD) study 2021 revealed that neurological disorders are now the top cause of disability-adjusted life years (DALYs) globally and are responsible for over 11 million deaths each year, highlighting the critical need to uncover the molecular pathways that underlie CNS disorders and the development of effective therapeutic targets.

A highly coordinated network of regulatory molecules and signaling pathways exists to regulate iron homeostasis, providing sufficient iron for proper protein synthesis and preventing iron-induced toxicity. Iron-regulatory proteins (IRPs) and iron-responsive elements (IREs) are involved in the post-transcriptional regulation of many iron metabolism-related genes, such as ferritin and transferrin receptor 1 (TfR1) (Kühn, 2015). Nuclear factor erythroid 2-related factor 2 (Nrf2) is a master regulator of the cellular antioxidant response, which regulates the expression of genes associated with both antioxidant defense and iron metabolism, such as heme oxygenase-1 (HO-1), ferritin heavy chain (FTH1), and solute carrier family 7 member 11 (SLC7A11) (He et al., 2020). Ferritinophagy is a selective autophagic process where ferritin is degraded to release the iron inside the cell, which can be used in redox reactions, and requires the involvement of another protein: the nuclear receptor co-activator 4 (NCOA4) (dos Santos Gonçalves, 2015; He et al., 2020). Other regulatory pathways involve the hepcidin-ferroportin axis responsible for the export of iron from the body and divalent metal transporter 1 (DMT1) and transferrin receptor involved in iron import into cells (Przybyszewska and Zekanowska, 2014; Vogt et al., 2021). Disruption of these well-regulated iron homeostatic mechanisms leads to increased iron accumulation within the cell, increased ROS generation, and a pro-oxidant intracellular environment that is pro-neurodegenerative and leads to ferroptosis of the neuronal cells. Moreover, recent studies have shown that dysregulated iron regulatory pathways also lead to increased inflammatory signaling in addition to iron-mediated ferroptotic cell death, thus setting the stage for a mechanistic cascade of iron, ferroptosis, and neuroinflammation in CNS diseases.

In addition to its role in regulated cell death, ferroptosis is seen as a key player in the pathogenesis of many central nervous system (CNS) diseases, as part of the umbrella of neuroinflammation (Xu et al., 2024). Ferroptosis is a unique mode of regulated cell death that involves iron-dependent lipid peroxidation arising from the dysfunction of antioxidant defense mechanisms, including the depletion of a key antioxidant, glutathione (GSH), inactivation of the antioxidant enzyme glutathione peroxidase 4 (GPX4), and the impairment of the iron-transporting cystine/glutamate antiporter (system Xc^–^), leading to irreversible membrane damage and neuronal death (Latunde-Dada, 2017; Su et al., 2019; Chen et al., 2021).

Recent studies show that ferroptosis and neuroinflammation do not occur independently, but that there is a self-reinforcing pathogenic vicious circle between them. Pro-inflammatory cytokines, Reactive Oxygen Species (ROS), and Nitric Oxide (NO) produced by activated microglia and astrocytes affect iron homeostasis, decrease antioxidant mechanisms, and increase lipid peroxidation, making neurons more susceptible to ferroptosis. In contrast, ferroptotic neurons secrete damage-associated molecular patterns (DAMPs), oxidized phospholipids and excess iron, all of which can lead to activation of pattern-recognition receptors, nuclear factor kappa B (NF-κB), and inflammasome signaling in the glial cells, thus sustaining chronic neuroinflammation and progressive neuronal degeneration (Li J. et al., 2022; Rauf et al., 2022).

The ferroptosis–neuroinflammation axis is an interesting target for therapeutic intervention because its bidirectional relationship has been identified as a key mechanism in neurodegenerative or neurovascular diseases, such as Alzheimer’s disease, Parkinson’s disease, multiple sclerosis, ischemic stroke and traumatic brain injury.

Current research suggests that the complex molecular networks connecting iron metabolism, ferroptosis, and neuroinflammation are not independent but are intricately linked in the pathogenesis of central nervous system illnesses (David et al., 2022). This is particularly essential for treatment since keeping iron levels stable and the ferroptotic cascade in check may stop neurons from dying and keep the inflammatory responses that cause disease in check (Plascencia-Villa and Perry, 2021). Thus, comprehending the interaction between iron control and neuroinflammatory signaling in central nervous system illnesses is essential for formulating comprehensive therapy strategies. This review examines recent progress in understanding iron homeostasis and its function in modulating ferroptosis and neuroinflammation in central nervous system neurodegenerative and neuroinflammatory illnesses. While there is significant experimental support that ferroptosis plays a role in the pathogenesis of CNS disorders, there are several unanswered questions. Ferroptosis seems to contribute to different disease types, stages, and cellular contexts to varying extents alongside other forms of regulated cell death, such as apoptosis, necroptosis, and pyroptosis. Furthermore, the results of some studies showed discrepancies in the biomarkers used to assess ferroptosis, and the differences in models and clinical results have hindered the translation of successful preclinical results into effective therapeutics. Taken together, these findings show that ferroptosis is a complex process and indicate that there is a need for a better understanding of the interplay between ferroptosis and neuroinflammatory signaling pathways. At the same time, researchers have focused more on alternative therapeutic approaches with natural products that can target oxidative stress, iron homeostasis, ferroptosis, and neuroinflammation all at once. The antioxidant, anti-inflammatory, and iron-regulating effects of bioactives including curcumin, resveratrol, epigallocatechin gallate (EGCG), and quercetin have been shown to involve modulation of the Nrf2, SIRT1, and NF-κB pathways. More recently, naturally derived compounds have been identified as modulators of multi-target and interconnected oxidative and inflammatory pathways, as summarized by Hui et al. (2024), and their therapeutic potential as such has been corroborated in CNS disorders. Still, the molecular mechanisms of the bidirectional relationship between ferroptosis and neuroinflammation are not fully understood. The processes are examined separately and are discussed in most of the present reviews, while the regulatory mechanisms and the translational importance of the integrated processes are only mentioned in a few of the existing reviews in a limited manner. Based on this, this review summarizes the current understanding of the mechanistic interaction between ferroptosis and neuroinflammation in CNS disorders, with a particular focus on the possible common molecular regulators such as SIRT1, Nrf2, NF-κB, COX-2, and iNOS, and introduces emerging therapeutic opportunities that may be useful in developing effective measures for preventing and treating neurological diseases such as neuroinflammation, including nutritional and natural-product-based approaches.

## 2 Ferroptosis and disorders of the central nervous system

### 2.1 Mechanism of ferroptosis

Ferroptosis is a type of regulated, iron-dependent cell death that is regulated and different from apoptosis, which involves excessive accumulation of lipid peroxides (Feng and Stockwell, 2018). Ferroptosis has distinctive morphological and biochemical characteristics that differentiate it from apoptosis, necrosis, and autophagy: small, dense mitochondria, loss of cristae or flattening of the mitochondrial cristae, disruption of the outer mitochondrial membrane, and the maintenance of nuclear morphology (Wang et al., 2020). Oxidative stress is the main cause of the process and is caused by overproduction of lipid reactive oxygen species (ROS) and inadequate detoxification (Di Giulio and Meyer, 2008). Ferroptosis differs from apoptosis, necroptosis, and pyroptosis in that it is primarily regulated by iron-dependent lipid peroxidation and not primarily by induction of caspases, by receptor-interacting protein kinase (RIPK) signaling, or by inflammasome activation to trigger inflammatory cell death (Table 1). This unique molecular signature has established ferroptosis as a distinct form of regulated cell death with important implications for neurodegenerative diseases, ischemic injury, and cancer.

**TABLE 1:** Comparison of ferroptosis with other major forms of regulated cell death.

Feature	Ferroptosis	Apoptosis	Necroptosis	Pyroptosis
Primary trigger	Iron-dependent lipid peroxidation	DNA damage, developmental signals	Death receptors, viral infection	Inflammasome activation
Major regulators	GPX4, system Xc^–^, ACSL4, LPCAT3	Caspase-3, Caspase-8, Bcl-2 family	RIPK1, RIPK3, MLKL	Caspase-1, Caspase-4/5/11, Gasdermin D
Iron dependence	**Yes**	No	No	No
Lipid peroxidation	Extensive	Minimal	Limited	Limited
ROS involvement	Central mechanism	Secondary	Moderate	Moderate
Mitochondria	Condensed, reduced cristae	Cytochrome c release	Swelling	Swelling
Plasma membrane	Rupture after lipid damage	Membrane blebbing	Membrane rupture	Gasdermin pores
Nuclear morphology	Preserved	Chromatin condensation	Preserved	Preserved
Inflammatory response	Moderate (DAMP-mediated)	Minimal	Strong	Very strong

**FIGURE 1:**
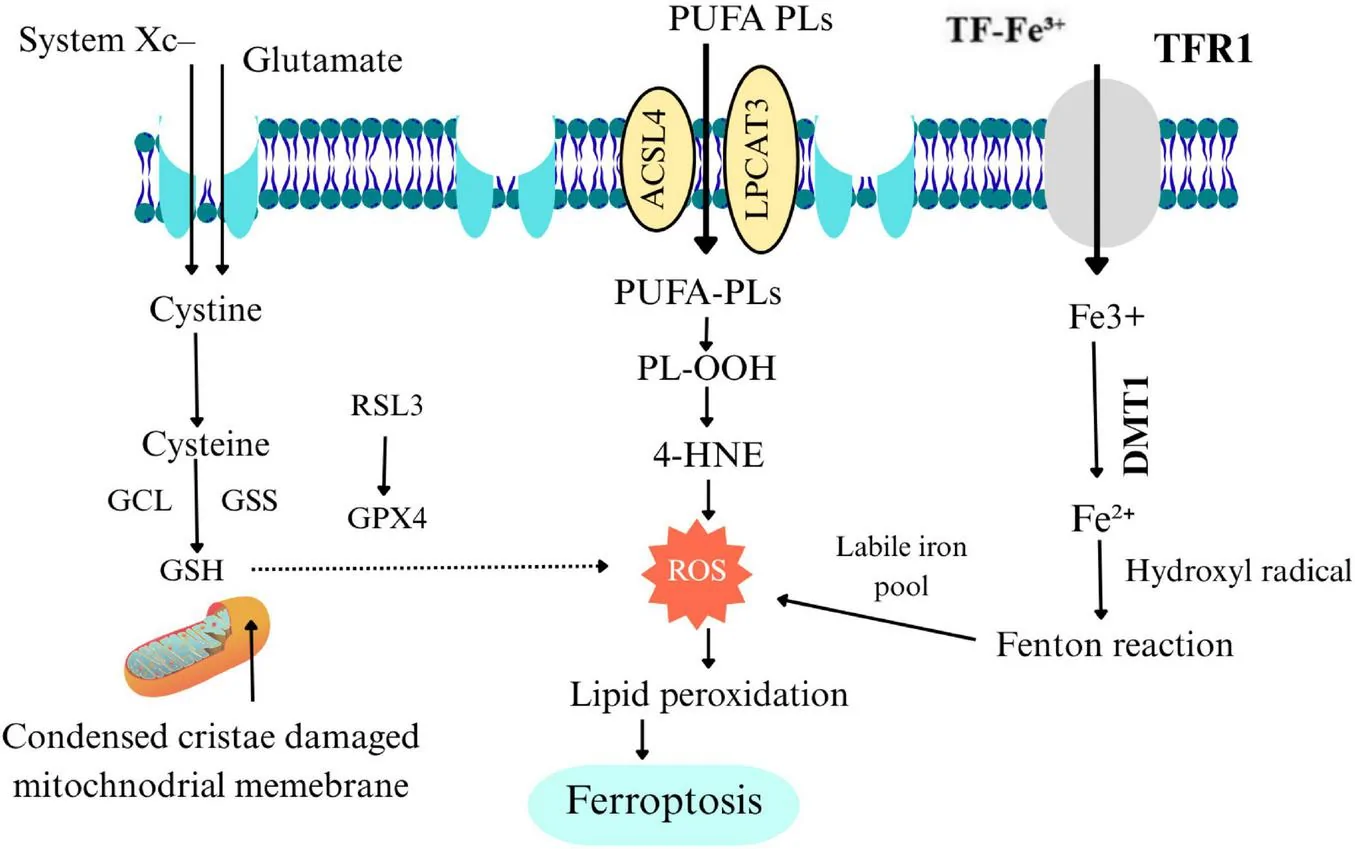
A schematic illustration of the molecular mechanism of ferroptosis, which is controlled by the GPX4-GSH axis and system Xc^–^ and involves iron metabolism, ROS build-up, and lipid peroxidation.

At the molecular level, ferroptosis starts with impaired antioxidant defenses, specifically those mediated by the antioxidant molecule glutathione (GSH), in cells (Kajarabille and Latunde-Dada, 2019). Glutathione peroxidase 4 (GPX4) is a selenoprotein that uses GSH as a cofactor and is a key enzyme that reduces phospholipid hydroperoxides into non-toxic lipid alcohols to prevent lipid peroxidation and to preserve membrane integrity (Forcina and Dixon, 2019). In the absence of GPX4, the presence of lipid hydroperoxides is not kept in check and leads to significant damage of membranes and eventually to ferroptotic cell death. The cystine/glutamate antiporter system Xc^–^ consists of two subunits, SLC7A11 and SLC3A2, that transport extracellular cystine into cells against the concentration gradient in exchange for glutamate. Cystine is then reduced to cysteine which is the rate limiting precursor for GSH biosynthesis (Figure 1). For instance, inhibition of system Xc^–^ (such as by erastin) results in the depletion of intracellular GSH, a decrease in GPX4 activity and therefore, in the promotion of ferroptosis (Liu et al., 2021; Li F. J. et al., 2022).

Mechanistic studies of the past years also show that the ferroptosis pathway is controlled by a complex network that goes beyond the canonical GPX4–system Xc^–^ axis. Cellular ferroptosis susceptibility is regulated by both lipid peroxide propagation pathways and phospholipid remodeling enzymes, as well as pathways for antioxidant defense and iron-dependent oxidoreductases. The membrane damage is especially exacerbated by selective oxidation of polyunsaturated phospholipids and by the lack of repair mechanisms, which leads to irreversible ferroptosis cell death. The results underscore that ferroptosis is a tightly regulated process that is linked with iron metabolism, lipid remodeling, and redox signaling, but is not just an oxidative stress response (Xing et al., 2024).

Iron metabolism is a key step in the onset and development of ferroptosis. The accumulation of intracellular ferrous iron (Fe^2+^) can lead to the generation of highly reactive hydroxyl radicals (●OH), one of the most harmful reactive oxygen species (ROS) in biological systems (Rochette et al., 2022) via the Fenton reaction, as shown below. Besides the Fenton reaction, ferrous iron (Fe^2+^) can be converted back to ferric iron (Fe3+) by superoxide anions (O2●-) which are mainly produced by the mitochondrial electron-transport chain, maintaining a continuous redox cycling of Fe. This self-propagating process leads to a significant increase in ROS production inside the cell, leading to an overload of the cellular antioxidant mechanisms, and to proteins, nucleic acids, and membrane lipids undergoing oxidative damage.

Polyunsaturated fatty acid (PUFA)-containing phospholipids are particularly susceptible to attack by hydroxyl radicals because of their bis-allylic hydrogen atoms. Abstraction of these hydrogen atoms by ROS leads to the formation of lipid radicals, which, on the addition of oxygen, result in the formation of lipid peroxyl radicals and lipid hydroperoxides. These chain reactions unfold quickly throughout cellular membranes, causing widespread phospholipid oxidation, loss of membranes, mitochondrial dysfunction, disruption of ion homeostasis and ultimately ferroptosis cell death.

The intracellular labile iron pool (Latunde-Dada, 2017) is tightly regulated by proteins involved in iron uptake, storage and export such as the transferrin receptor (TfR1), ferritin and ferroportin (FPN). The dysregulation of any of these iron handling proteins leads to an excess of catalytically active Fe^2+^, which boosts ROS production and renders cells vulnerable to ferroptosis. This means iron homeostasis is crucial to reduce oxidative stress and maintain the viability of the neurons. Excessive accumulation of iron inside the cell (Xie et al., 2016) disrupts this delicate balance and makes the cells more vulnerable to ferroptosis.

Lipid metabolism is a key determinant of cellular vulnerability to ferroptosis. Acyl-CoA synthetase long-chain family member 4 (ACSL4) and lysophosphatidylcholine acyltransferase 3 (LPCAT3) are enzymes that convert the preferred substrates for lipid peroxidation, polyunsaturated fatty acids (PUFAs), into membrane phospholipids (Jalil et al., 2019). These PUFA-containing phospholipids are then oxidized by lipoxygenases (LOXs) and other iron-dependent oxidoreductases, leading to the production of phospholipid hydroperoxides, which further drive ferroptotic signaling (Ma et al., 2022). New findings have shown that lipid remodeling is not only a downstream effect of oxidative stress, but it is a key factor in sensitivity to ferroptosis. Cellular redox vulnerability is determined by the phospholipid composition of cellular membranes, the level of PUFA enrichment, and the ratio of lipid peroxidation to antioxidant repair. Cells that are enriched with PUFA-phospholipids that are oxidizable are more vulnerable to ferroptosis, while enhanced phospholipid repair and antioxidant defense mechanisms are associated with resistance to lipid peroxide accumulation. Moreover, coordinated regulation of ACSL4, LPCAT3, GPX4, and membrane phospholipid remodeling has come to light as a major molecular axis that regulates ferroptosis commitment in a broad range of pathological conditions, including neurodegenerative diseases (Ma et al., 2026).

Dysregulated lipid metabolism is a strong contributor to oxidative damage of the membranes and to the acceleration of ferroptosis cell death in neuronal cells, which have lipid-rich membranes and high oxygen consumption. The results also highlight the importance of lipid changes in addition to iron deposition and antioxidant status, as they are the core biochemical processes responsible for ferroptosis and can be potential therapeutic targets in CNS diseases.

It can be concluded that ferroptosis is caused by a breakdown in redox balance, by a build-up of intracellular iron, and by the peroxidation of membrane phospholipids that are rich in polyunsaturated fatty acids (Mortensen et al., 2023). Iron metabolism, lipid remodeling and antioxidant defense pathways are linked to the vulnerability of the cell to ferroptosis injury and may explain the mechanisms of disease progression in many pathological conditions. Such knowledge of these molecular mechanisms has significant therapeutic value in cancer, neurodegenerative disease, ischemia–reperfusion injury and other diseases in which ferroptosis plays a role in disease development. Thus, therapeutic approaches that target the key regulators of ferroptosis may offer new prospects in disease prevention and treatment.

### 2.2 Hepcidin, NCOA4, PCBPs, the IRP/IRE system, and DMT1 for iron homeostasis

Iron is a vital micronutrient for numerous biological processes, encompassing DNA synthesis, mitochondrial respiration, oxygen transport, and enzyme functions (Theil and Goss, 2009). Iron can move from one form to another; therefore, it’s crucial to maintain iron levels in check to minimize the oxidative stress that free iron can cause (Puntarulo, 2005). In mammals, iron-regulatory proteins and signaling pathways work together in extremely complicated ways to keep iron levels in cells and throughout the body in balance (Eisenstein, 2000). Various clinical illnesses have been linked to disruptions in this regulating process, including neurodegenerative disorders marked by simultaneous inflammation, oxidative stress, and iron build-up. Hepcidin, nuclear receptor co-activator 4 (NCOA4), poly(rC)-binding proteins (PCBPs), the iron-regulatory protein/iron-responsive element (IRP/IRE) system, and divalent metal transporter 1 (DMT1) are important parts of this process (Luscieti, 2016). Each portion has its own technique of keeping iron levels consistent inside cells and throughout the body.

#### 2.2.1 Hepcidin- the iron regulator

Hepatocytes are the major cells that create hepcidin, a 25-amino acid peptide hormone that is made when the body needs red blood cells, is inflamed, has low iron levels, or has low oxygen levels (Rauf et al., 2020). It is the primary regulator of systemic iron homeostasis, controlling the amount of iron absorbed from food and released by hepatocytes and macrophages. Hepcidin binds to ferroportin 1 (FPN1), the sole iron exporter found on the basolateral membrane of placental syncytiotrophoblasts, enterocytes, and macrophages (Wang, 2007). The interaction between hepcidin and FPN1 leads cells to take in FPN1 and lysosomes to break it down. This keeps iron from getting out of the cells and into the blood (Wang, 2007; De Domenico et al., 2011; Gao et al., 2019).

In inflammatory situations, interleukin-6 (IL-6) activates the JAK/STAT3 pathway to make hepcidin. This response to iron sequestration lowers blood iron levels (hypoferremia) to inhibit the spread of infections. This causes a functional iron deficiency and anemia of inflammation (Lanser et al., 2021). Hepcidin levels that stay high for a long time, which is typical in chronic inflammatory and neurodegenerative diseases, make it harder for cells to obtain iron for metabolism (Arosio, 2022). This rise, strangely, leads to iron buildup, oxidative stress, and ferroptosis, especially in brain endothelial and glial cells (Zhao et al., 2023).

#### 2.2.2 Ferritinophagy and NCOA4: altering the method of iron storage in cells

The protein ferritin is composed of 24 subunits, including light (FTL) and heavy (FTH1) chains. It is the major place in the cell where iron is stored (Muhoberac and Vidal, 2019). It can inhibit iron from causing oxidative damage by holding onto up to 4,500 iron atoms in a way that does not change. Ferritinophagy, the controlled breakdown of ferritin by autophagy, depends on the nuclear receptor co-activator 4 (NCOA4), a selective cargo receptor (Ravi, 2019; Dorman, 2022).

When NCOA4 attaches to ferritin in the cytosol and transfers it to autophagosomes for lysosomal degradation, it releases bioavailable ferrous iron (Fe^2+^) into the cytoplasm (Santana-Codina and Mancias, 2018). This iron pool is helpful for many metabolic activities, but too much of it can produce the Fenton reaction, which makes ROS. When NCOA4 is turned up, cells are more likely to die from ferroptosis because it makes iron more available inside the cell and speeds up the turnover of ferritin (Santana-Codina et al., 2021). On the other hand, removing or blocking NCOA4 keeps ferritin storage intact, which lowers free iron levels and makes cells more resistant to ferroptotic cell death (Bengson, 2020).

Increasingly, NCOA4 mediated ferritinophagy has been linked to the pathogenesis of neurodegenerative disorders by its role in regulating the intracellular iron homeostasis and ferroptotic susceptibility. Neurons and glia cells perform excessive ferritinophagy that leads to an increase of labile iron pool, which then stimulates Fenton chemistry, lipid peroxidation, mitochondrial dysfunction and oxidative stress (Sui et al., 2025). Recent studies indicate that the dysregulation of NCOA4 activity leads to persistent microglial activation and neuroinflammation through the augmentation of iron-driven generation of ROS and secretion of pro-inflammatory mediators. In addition, hyper-degradation of ferritin can promote neuronal ferroptosis in diseases like Alzheimer’s disease, Parkinson’s disease, and other neurodegenerative disorders associated with iron mislocalization in the brain. In contrast, blocking NCOA4/ferritinophagy can maintain intracellular ferritin levels, decrease the labile iron pool, inhibit lipid peroxidation, and protect neurons from ferroptosis-induced degeneration, suggesting that ferritinophagy is a potential therapeutic target to reduce ferroptosis-associated neurodegeneration. Therefore, the modulation of NCOA4 activity may be a therapeutic strategy to restore iron homeostasis and reduce iron-related injury to neurons under the circumstances of iron dysregulation.

#### 2.2.3 Poly (rC)-binding proteins (PCBPs): control of enzymatic cofactors and iron chaperones

PCBPs are proteins that bind to RNA and perform a variety of functions. They are also known as iron chaperones because they help transfer iron into cells, especially PCBP1 and PCBP2 (Subramanian, 2013). PCBPs attach to cytosolic Fe^2+^ and transport it to other proteins, including those that create iron-sulfur clusters, ferritin, and iron-dependent enzymes such as prolyl hydroxylases (Yanatori et al., 2019).

PCBP1 is the one who decides how big the labile iron pool is. It transports iron to ferritin for storage. On the other hand, PCBP2 only transfers iron to enzymes in the nucleus and cytoplasm that need it as a cofactor (Table 2). When PCBP1 stops working, it raises free iron levels and lowers ferritin iron loading, which causes oxidative stress and lipid peroxidation. Too much PCBP2, on the other hand, can improve iron metabolism, but it may also make people more likely to suffer ferroptosis since it makes more iron available for producing ROS (Protchenko et al., 2021).

**TABLE 2:** Iron metabolism regulators: gatekeepers of ferroptosis in CNS disorders.

Regulator	Primary function	Regulatory mechanism of iron	Effects on ferroptosis	Significance in the CNS disorders
Hepcidin	Principal hormone governing systemic iron regulation	Interacts with FPN1 (ferroportin), leading to its internalization and degradation, thereby restricting iron export.	Elevated hepcidin levels lead to intracellular iron accumulation, reactive oxygen species (ROS) generation, and ferroptosis.	Chronic neuroinflammation increases hepcidin levels, leading to iron overload and neurodegeneration.
NCOA4	Mediator of ferritinophagy	Facilitates the transport of ferritin to autophagosomes for degradation, resulting in the release of bioavailable Fe^2+^.	Enhances the labile iron pool, facilitating lipid peroxidation and ferroptosis; inhibition provides protective effects.	Neuroinflammation, mitochondrial failure, and microglial activation are all associated with excessive NCOA4 activity.
PCBPs	Cytosolic iron chaperones	PCBP1 facilitates the delivery of Fe^2+^ to ferritin for storage, while PCBP2 is responsible for transferring Fe^2+^ to iron-dependent enzymes.	Free iron is increased by PCBP1 deficit, while ROS production and ferroptosis risk are increased by PCBP2 overexpression	PCBP dysfunction reduces antioxidant capacity and neural energy, making CNS cells susceptible to ferroptosis.
IRP/IRE system	Post-transcriptional iron metabolism gene regulation	TfR1, DMT1, FPN1, and ferritin translation are all regulated by binding IREs in mRNAs based on iron status.	Iron overload, the production of ROS, and ferroptotic mortality are caused by dysregulated IRP activity in iron-rich environments	Neurodegeneration is associated with IRP2 deficit; cellular iron buffering is disrupted by changed IRP/IRE dynamics.
DMT1	Iron importer	After transferrin absorption, it transports Fe2+ from endosomes and across plasma membranes.	Intracellular iron and ferroptosis susceptibility are increased by DMT1 overexpression in hypoxic/inflammatory conditions.	During CNS inflammation, mitochondrial DMT1 is overexpressed in glia and neurons, promoting ROS and organelle iron loading

PCBPs help neurons and glial cells in the central nervous system deal with iron (Kulaszyñska et al., 2024). Iron is necessary for neurons to work properly since they have a high metabolic need. If PCBP-mediated iron trafficking isn’t operating properly, it might make antioxidant defenses, mitochondrial function, and cellular energetics weaker, which would make them more prone to being affected by ferroptosis (Lane et al., 2015).

### 2.2.4 IRP/IRE system-mediated post-transcriptional control of iron homeostasis

After transcription, one important method for regulating iron levels in cells is the IRP/ IRE system. IRP1 and IRP2 are proteins that bind to RNA and modify how stable or translated mRNAs are when they have IREs in their untranslated regions (UTRs) (Kühn, 2015; Zhou and Tan, 2017).

When iron levels are low, IRPs bind to IREs in the 5’-UTR of ferritin and ferroportin mRNAs. This prevents translation and makes it harder to store and move iron (Wilkinson, 2022). They stabilize and improve iron absorption by binding to IREs in the 3’-UTR of DMT1 and TfR1 mRNAs at the same time. Too much iron, on the other hand, hinders IRP from binding. This makes ferritin production go up and iron import go down (Pantopoulos, 2004).

This feedback loop is quite accurate, so cells can swiftly adjust to variations in iron levels. But diseases like neurodegeneration or long-term inflammation can make IRP/IRE regulation less stable. In iron-replete environments, dysregulated IRP activation may facilitate excessive iron absorption and reduced sequestration, potentially leading to ferroptosis and oxidative injury (Zhang et al., 2014). Moreover, in animal models, the lack of IRP2 has been linked to neurodegeneration, underscoring its critical function in neuronal iron homeostasis (Liang et al., 2025).

#### 2.2.5 Iron entry via divalent metal transporter 1 (DMT1)

DMT1 is a transmembrane protein that transports divalent metal ions, such as Fe^2+^, from the exterior of the cell into the cytoplasm (Garrick et al., 2003). It has two jobs: it helps the duodenum take in iron from food, and it helps iron get out of endosomes after transferrin receptor-mediated endocytosis (Garrick et al., 2006).

Low oxygen levels, inflammation, and iron levels have a big effect on how much DMT1 is made. When there isn’t enough iron or oxygen, HIF-1α and IRP/IRE signaling make DMT1 levels go up (Doguer, 2016). In central nervous system disorders, neurons and glial cells subjected to oxidative stress or inflammatory cytokines exhibit elevated levels of DMT1. This up-regulation induces lipid peroxidation and ferroptosis, which leads to greater iron accumulation and increased ROS production (Urrutia et al., 2013).

DMT1 contains multiple isoforms, and some of these modifications happen in certain areas of cells, including the mitochondria. When mitochondrial DMT1 is not working properly, it raises oxidative stress in the mitochondria and encourages the organelle to take in iron. This, in turn, speeds up the production of heme and iron-sulfur clusters (Onukwufor et al., 2022).

Hepcidin, NCOA4, PCBPs, the IRP/IRE system, and DMT1 all work together to keep the balance of iron intake, storage, usage, and export. Changes in these regulators increase oxidative stress, upset iron homeostasis, and make the labile iron pool bigger, making cells more likely to die from ferroptosis (Table 2). This type of imbalance contributes to the pathogenesis of numerous neurodegenerative and neuroinflammatory disorders within the central nervous system (CNS), where iron metabolism is intricately associated with neuronal integrity and glial activity. A thorough understanding of these molecular entities enables targeted therapeutic interventions aimed at restoring iron homeostasis and reducing ferroptotic cell death, while concurrently offering substantial insights into the mechanisms underlying iron-induced neurotoxicity.

## 3 Ferroptosis in central nervous system disorders

**FIGURE 2:**
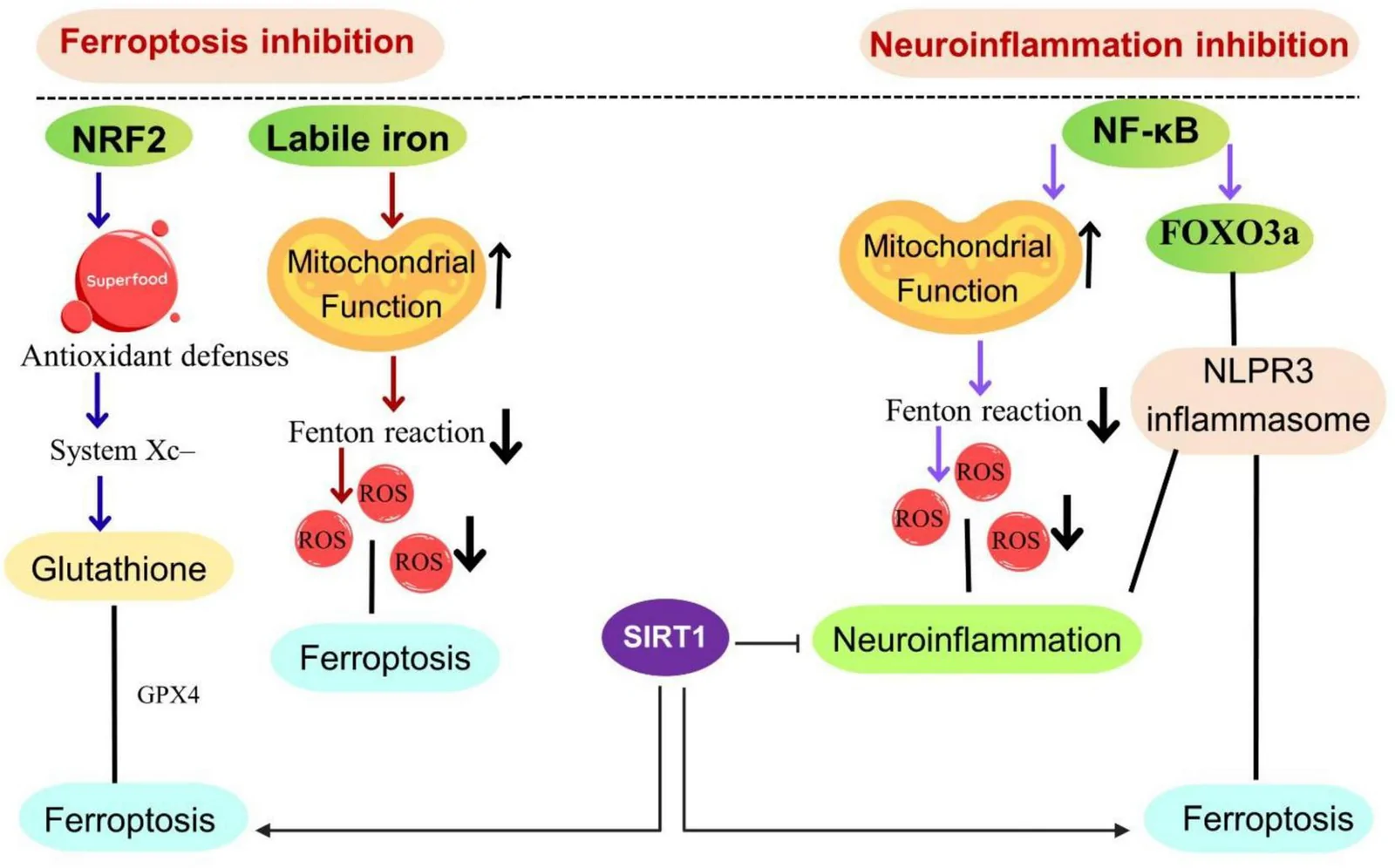
A schematic illustration of how SIRT1 halts ferroptosis and neuroinflammation by controlling NRF2, NF-κB, FOXO3a, labile iron, mitochondrial activity, and oxidative stress pathways.

Ferroptosis is a regulated form of cell death defined by the accumulation of lipid peroxide in the presence of iron and the excessive generation of reactive oxygen species (ROS), and is responsible for many CNS diseases (Wu et al., 2018). Ferroptosis causes damage to the membranes and neuronal death by redox imbalance via iron metabolism, glutathione (GSH) depletion, and lipid peroxidation (Wang et al., 2023). Because of their high PUFA content, their increased consumption of oxygen, and relatively low antioxidant defenses, neurons are especially susceptible to ferroptosis-induced damage (Figure 2). Experimental and clinical findings in the last few years suggest that ferroptosis plays a role in both chronic and acute CNS diseases via disease-specific molecular mechanisms involving iron dysregulation, antioxidant deficiency, mitochondrial dysfunction and neuroinflammation. As a result, ferroptosis is a shared mechanism of death in Alzheimer’s disease, Parkinson’s disease, Huntington’s disease, ischemic stroke, traumatic brain injury, epilepsy and brain tumors, which all have different causes. Based on the growing body of science and clinical evidence, ferroptosis is thus a promising therapeutic target for various CNS diseases (Liu et al., 2024).

AD involves the progressive loss of cognitive ability, with the presence of amyloid-β deposition, hyperphosphorylated tau, oxidative stress, and chronic neuroinflammation. Excessive iron accumulation in the hippocampus and cerebral cortex, lipid peroxidation, mitochondrial dysfunction, and antioxidant defense depletion have been identified as key factors that trigger ferroptosis in the pathogenesis of AD (Reichert et al., 2020; Kuang et al., 2020). Iron accumulation in the cells increases the production of hydroxyl radicals by the Fenton reaction, which causes phospholipid oxidation and the generation of toxic lipid peroxidation products like MDA and 4-hydroxynonenal (4-HNE) that worsen the neuronal damage and inflammatory response (Ayala et al., 2014). Therapeutic studies in the past few years have pointed toward receptor-biased signaling strategies that downregulate and inhibit multiple pathways of neuroinflammation, oxidative stress, and ferroptosis, and thus provide disease-modifying avenues of intervention in Alzheimer’s disease (Wang et al., 2025).

The association of Parkinson’s disease (PD) with ferroptosis is evident due to the excessive accumulation of iron in substantia nigra, mitochondrial dysfunction, α-synuclein aggregation, and impaired antioxidant defenses (Reichert et al., 2020). Reactive oxygen species generation and lipid peroxidation is increased by iron overload, while depletion of glutathione and reduction in GPX4 activity increases dopaminergic neurons’ susceptibility to ferroptotic cell death (Conrad and Sato, 2012; Lewerenz et al., 2013). Pharmacological modulation of ferroptosis has also been shown to help preserve the survival of dopaminergic neurons by reestablishing redox homeostasis, inhibiting lipid peroxidation, and decreasing neuroinflammation in recent studies. Emerging trends in the development of ferroptosis-targeted therapeutics such as metal-based and redox-modulating compounds have demonstrated their neuroprotective activity in experimental models of Parkinson’s disease, making ferroptosis a promising therapeutic target (Meng et al., 2026).

Additionally, abnormal iron accumulation in striatal regions, mitochondrial dysfunction, oxidative stress, and a deficit in cell antioxidant capacity are associated with Huntington’s disease (HD) and ferroptosis (Reichert et al., 2020). Raised lipid peroxidation and glutathione depletion make neurons more vulnerable to ferroptosis-related cell death, which can contribute to the progressive neurodegeneration. While the therapeutic importance of ferroptosis modulation in HD is still being actively explored, experimental evidence suggests that the modulation of iron homeostasis and antioxidant defenses could be an interesting opportunity to slow the progression of the disease (Table 3).

**TABLE 3:** Comparative characteristics of ferroptosis in major neurodegenerative disorders.

Feature	Alzheimer’s disease	Parkinson’s disease	Huntington’s disease
Primary affected brain region	Hippocampus and cerebral cortex	Substantia nigra pars compacta	Striatum
Iron dysregulation	Cortical and hippocampal iron accumulation	Excess iron deposition in substantia nigra	Striatal iron accumulation
Major pathological features	Amyloid-β plaques, tau hyperphosphorylation	α-Synuclein aggregation, dopaminergic neuronal loss	Mutant huntingtin protein aggregation
Major ferroptosis mechanisms	Lipid peroxidation, GPX4 dysfunction, oxidative stress	Iron overload, mitochondrial dysfunction, GPX4 depletion	Iron-mediated oxidative stress, mitochondrial dysfunction, impaired antioxidant defenses
Representative ferroptosis biomarkers	GPX4↓, GSH↓, MDA↑, 4-HNE↑	GPX4↓, lipid ROS↑, iron↑	Iron↑, ROS↑, GSH↓
Potential therapeutic strategies	Iron chelators, GPX4 activation, receptor-biased therapies	Ferroptosis inhibitors, redox modulators, iron chelation	Restoration of iron homeostasis and antioxidant defenses

Ferroptosis is also an important cause of acute CNS injury, such as ischemic stroke (Qin et al., 2021). Reactive oxygen species (ROS) are produced in abundance during cerebral ischemia and reperfusion, which quickly outpaces the brain’s antioxidant capacity, causing glutathione depletion, iron accumulation, mitochondrial dysfunction, and many other changes (Schaller and Graf, 2004; Tang et al., 2020). The pathological events all contribute to lipid peroxidation and ferroptosis-mediated neuronal death. In all experimental stroke models, there is a correlation between increased iron deposition and lipid peroxidation with infarct progression and neurological dysfunction. Moreover, iron chelators like deferoxamine and ferroptosis inhibitors including ferrostatin-1 and liproxstatin-1 have been demonstrated to be effective at lowering infarct size, oxidative damage, neurological deficits and lipid peroxidation in pre-clinical studies (Guo et al., 2023; Pan et al., 2023). In addition to GPX4 as a key player in ischemic neuronal injury, pharmacological interventions that help restore GPX4 activity and intracellular glutathione levels further support the role of ferroptosis. The critical role that GPX4 plays in ischemic neuronal injury is further underscored by pharmacological interventions that restore GPX4 activity and cellular glutathione levels.

Ferroptosis is one of the secondary injuries following the primary mechanical damage, which occurs after traumatic brain injury (TBI) (Qin et al., 2021). The disruption of the blood-brain barrier, mitochondrial dysfunction, the degradation of hemoglobin, and the intracellular release of iron establish an oxidative microenvironment which strongly supports ferrthatoptotic neuronal death. The experimental TBI models are characterized by glutathione depletion, a decrease in GPX4 activity, increased lipid peroxidation, and mitochondrial morphology changes such as condensed mitochondria with decreased cristae observed in the pericontusional brain tissue (El-Gazar et al., 2024). Hence, ferroptosis is a promising therapeutic target as the pharmacological inhibition of lipid peroxidation and iron accumulation is shown to significantly enhance neurological recovery in experimental models (Fang et al., 2023). Importantly, there is growing clinical evidence that markers of ferroptosis, such as lipid peroxidation products, changes in iron metabolism and decreases in antioxidant capacity, are linked to the severity of injuries and neurological outcomes after TBI. These findings suggest that ferroptosis-associated markers could be used clinically for disease monitoring and patient stratification and that ferroptosis-targeted therapies could serve as an exciting add-on to current treatment strategies to reduce secondary brain injury and promote long-term functional recovery (Table 4).

**TABLE 4:** Representative ferroptosis-associated biomarkers across central nervous system disorders.

CNS disorder	Major ferroptosis biomarkers	Clinical significance	Potential therapeutic implication
Alzheimer’s disease	Iron accumulation, GPX4↓, GSH↓, MDA↑, 4-HNE↑	Progressive neuronal degeneration and cognitive decline	Iron chelation, GPX4 restoration, antioxidant therapy
Parkinson’s disease	Substantia nigra iron↑, GPX4↓, lipid peroxidation↑, α-synuclein-associated oxidative stress	Dopaminergic neuronal degeneration	Ferroptosis inhibitors, iron chelators, redox modulators
Huntington’s disease	Striatal iron accumulation, ROS↑, GSH↓	Progressive neuronal loss	Antioxidants and iron homeostasis restoration
Ischemic stroke	Lipid peroxidation↑, GPX4↓, iron overload	Infarct progression and neurological deficits	Ferrostatin-1, Liproxstatin-1, Deferoxamine
Traumatic brain injury	Iron accumulation, MDA↑, GPX4↓, GSH↓	Secondary brain injury severity	Anti-ferroptotic therapy and biomarker-guided treatment
Epilepsy	Hippocampal iron↑, ROS↑, GPX4↓	Seizure-associated neuronal injury	Ferroptosis inhibition and antioxidant therapy
Glioma	ACSL4↑, lipid ROS↑, GPX4 dysregulation	Tumor progression and treatment response	Ferroptosis-inducing anticancer therapies

Epilepsy has also been linked to ferroptosis, which involves frequent seizures causing excessive glutamate release, oxidative stress, neuroinflammation, and iron homeostasis disruption. Increased seizure activity in the brain leads to the activation of NADPH oxidases and lipoxygenases, as well as a rise in the intracellular calcium level, which stimulates the production of ROS and lipid peroxidation (Sun et al., 2022). Iron accumulation in the hippocampus and decreased levels of glutathione and GPX4 have been linked to both epilepsy patients and experimental seizure models, indicating that ferroptosis may play a crucial role in seizure-related neuronal damage. Ferroptosis inhibitors, therefore, show potential therapeutic promise by exerting antioxidant effects, maintaining neuronal viability, and reducing seizure severity and frequency (Su et al., 2024).

While ferroptosis is mostly related to the onset of neurodegenerative diseases, the involvement of ferroptosis in primary brain tumors, including gliomas, is gaining more attention. In contrast to neurodegenerative disorders, where ferroptosis plays a role in neuronal loss, acute induction of ferroptosis is thought to be therapeutic in glioma due to selective killing of tumor cells. Recent research has shown that pharmacological induction of ferroptosis would inhibit the proliferation of gliomas, increase sensitivity to chemotherapy, and reduce resistance to chemotherapy by targeting iron metabolism, lipid peroxidation, and antioxidant defense pathways. The results suggest that ferroptosis induction is a potential therapeutic approach for the treatment of gliomas and warrant clinical evaluation of ferroptosis combination therapies (Li et al., 2026).

Poor iron metabolism, redox imbalance, and lipid peroxidation define the ferroptotic pathway in CNS disorders. GPX4, Nrf2, and ferritinophagy control ferroptosis (Latunde-Dada, 2017). NCOA4-mediated ferritin degradation increases ferroptosis risk by releasing free iron. Elevated inflammatory mediators such as IL-6, TNF-α, and IL-1β in central nervous system illnesses might increase ferroptotic cell death by causing oxidative stress or impairing antioxidant defenses (Guo et al., 2024). Ferroptosis and inflammation form a negative cycle when lipid peroxidation products like 4-HNE activate pattern recognition receptors and maintain neuroinflammation (Cheng et al., 2021).

These discoveries have important therapeutic implications. Modification of ferroptosis decreases neuronal death and neuroinflammation in CNS disorders. Neuroprotection is being explored with iron chelators, GPX4 activators, glutathione precursors (such as N-acetylcysteine), and lipid peroxidation inhibitors (Tan et al., 2024). The ability to understand the upstream signaling pathways that regulate ferroptosis, such as p53, Nrf2, and ACSL4, could be useful for the development of new therapeutic targets. Recent studies additionally reveal the potential for receptor-targeted therapeutic approaches to simultaneously regulate oxidative stress, neuroinflammation, and ferroptosis, suggesting a new therapeutic space for ferroptosis interventions, especially in the context of Alzheimer’s disease (Wang et al., 2025). Nevertheless, treatment translation still needs the availability of relevant ferroptosis markers, better BBB permeability, and the rigorous validation of novel ferroptosis-targeted treatments in the clinic (Wan et al., 2023).

Ferroptosis is a vital molecular connection between oxidative stress, iron dysregulation, lipid peroxidation, and neuroinflammation in both chronic and acute CNS pathologies. Because of its role in neuronal injury and progression of disease in various neurological disorders, ferroptosis emerges as a promising therapeutic target for these disorders. Future studies should aim to establish effective ferroptosis-specific biomarkers, understand its regulatory mechanisms in different diseases, optimize the delivery of ferroptosis-targeted medicines into the blood-brain barrier, and validate these approaches in well-designed clinical trials. Additionally, a combination of ferroptosis modulation and existing neuroprotective and anti-inflammatory drugs may offer synergistic effects and expedite the translation of experimental results to clinical application. Further study of the molecular interaction between ferroptosis and other mechanisms of regulated cell death will make it easier to develop effective treatments for CNS diseases.

**FIGURE 3:**
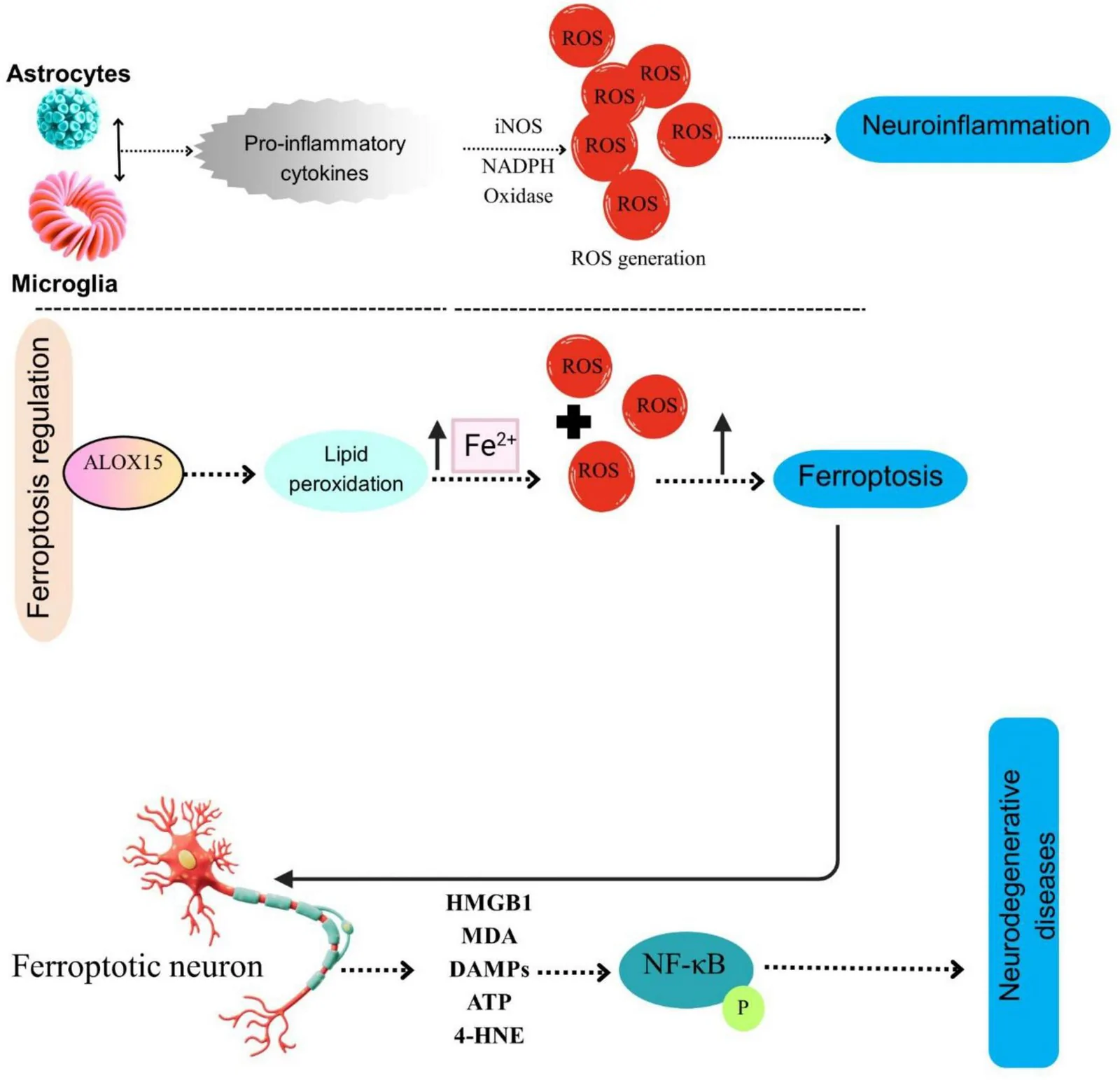
A schematic illustration of the NF-κB signaling pathway linking oxidative stress to neuroinflammation and ferroptosis through a feedback loop.

Neuronal dysfunction and neurodegeneration are common in CNDs like Alzheimer’s, Parkinson’s, and multiple sclerosis, along with acute traumas like ischemic stroke and TBI (Zehravi et al., 2022). The primary components of neuroinflammation are glial activation and pro-inflammatory mediators, as well as ferroptosis, a controlled cell death brought on by lipid peroxidation (Figure 3). Recent discoveries reveal that ferroptosis and neuroinflammation form a pathogenic feedback loop that enhances neuronal damage and accelerates disease development (Li et al., 2024).

Iron-dependent lipid peroxides build up in polyunsaturated phospholipid membranes due to ferroptosis. ROS oxidize these lipids, especially with redox-active iron, speeding Fenton and Haber-Weiss processes (Endale et al., 2023). Normally, antioxidant defenses, especially the GSH-GPX4 pathway, decrease lipid peroxidation and ferroptotic cell death. GSH helps GPX4 convert lipid hydroperoxides into harmless lipid alcohols (Maiorino et al., 2018). CND-related disorders change intracellular GSH, GPX4, and iron homeostasis. Changes create an oxidative environment suited for ferroptosis. Neurons are weak due to their high metabolic rate, many mitochondria, few antioxidants, and polyunsaturated fatty acids in their cell membranes (Su et al., 2019; Lin et al., 2021).

Neuroinflammation is the normal reaction of the immune system of the CNS. Microglia and astrocytes are involved in response to infections, traumas, and neurodegeneration. Acute neuroinflammation enhances tissue repair and clearing of debris, but excessive inflammation can result in side effects leading to the production of ROS, NO, and pro-inflammatory cytokines including TNF-α, IL-1β, and IL-6, which may cause tissue damage (Rauf et al., 2022). Microglia are highly plastic cells that can be activated either in a pro-inflammatory (M1) or anti-inflammatory and tissue reparative (M2) state depending on the microenvironment they are exposed to. Excessive production of ROS, nitric oxide, and TNF-α, IL-1β, and IL-6 by M1-polarized microglia leads to oxidative stress, iron dysregulation, and ferroptotic neuronal injury. M2-polarized microglia, on the other hand, promote tissue repair through the production of anti-inflammatory cytokines like IL-10 and TGF-β, and promote the clearance of debris, which reduces oxidative stress and ferroptosis susceptibility. Modulation of microglial polarization is an effective therapeutic strategy to break the vicious cycle of chronic neuroinflammation and neurodegenerative processes, and an imbalance favoring persistent M1 polarization has been linked to chronic neuroinflammation and progressive neurodegeneration. These mediators directly damage neurons and activate ferroptotic pathways leading to oxidative stress and iron dysregulation (Li J. et al., 2022).

Ferroptosis and neuroinflammation are connected by oxidative stress. This can cause and affect both. Through NOX and iNOS, active microglia produce a lot of ROS and NO in neuroinflammation (Simpson and Oliver, 2020; Qu et al., 2022). These reactive chemicals peroxidize neuronal membranes, enabling ferroptosis. Inflammatory cytokines adjust cell ferroptosis risk by lowering the cystine/glutamate antiporter system Xc^–^, which transports cystine into cells (Table 5). GSH has cysteine (Ashraf et al., 2020). Blocking system Xc^–^ leads to reduced GSH production, GPX4 dysfunction, and uncontrolled lipid hydroperoxide buildup. Ferroptosis in neurons increases with inflammation (Dar et al., 2024).

**TABLE 5:** Crosstalk between ferroptosis and neuroinflammation in central nervous system disorders.

Feature	Ferroptosis	Neuroinflammation	Interaction and feedback loop	Implications for CNS conditions
Initiating factors	Lipid peroxidation, iron buildup, GSH deficiency, and GPX4 inactivation	Misfolded proteins, infection, trauma, DAMPs, and cytokines	Iron, ROS, and lipid peroxidation augment both routes and inflammation.	Ischemic stroke, AD, PD, MS, TBI, and epilepsy
Major molecular triggers	Fenton reaction, ACSL4, system Xc^–^ inhibition, and PUFA oxidation	NF-κB activation, TNF-α, IL-1β, IL-6, iNOS/NO●, and NLRP3 inflammasome	Cytokines increase ferroptosis by blocking system Xc^–^ and GPX4. Ferroptotic cells emit DAMPs, triggering microglia.	Self-replicating cycles increase neuronal death and oxidative damage.
Redox mediators	Lipid ROS, 4-HNE, MDA, and hydroxyl radicals	ROS, NADPH oxidases, mitochondrial dysfunction, and RNS (NO●, ONOO^–^)	Oxidative stress from inflammation promotes lipid peroxidation. Ferroptosis increases redox imbalance.	drives ischemia episodes and subsequent damage following TBI
Affected key cells	PUFA-rich membranes and a limited antioxidative capacity primarily characterize neurons.	Microglia and astrocytes serve as immune responders, while neurons function as targets.	Ferroptosis in neurons stimulates glia, and glial-derived ROS and cytokines make ferroptosis worse.	Glial scarring, neurodegeneration, BBB damage
Mediators released	HMGB1, ATP, oxidized lipids, and 4-HNE are significant molecules in cellular processes.	Prostaglandins, NO, TNF-α, IL-1β, and IL-6	Ferroptotic DAMPs interact with TLRs and NLRs, leading to the activation of NF-κB and inflammasomes.	Maintains persistent inflammation and hastens the advancement of neurodegenerative processes.
Protective pathways	Nrf2-GPX4 axis, SLC7A11 (system Xc^–^), and the suppression of ferritinophagy	Anti-inflammatory cytokines (IL-10), resolution signals, SIRT1/Nrf2 activation	Ferroptosis and inflammation are both regulated by Nrf2 and SIRT1	The cycle can be broken by therapeutic modification
Experimental evidence	N-acetylcysteine, iron chelators, and ferroptosis inhibitors (such as ferrostatin-1 and liproxstatin-1) increase neuronal survival.	Ferroptotic damage and oxidative stress are lessened by anti-inflammatory medications	Combination treatments demonstrate additive neuroprotection in CNS damage animal models.	Dual-targeted therapies have strong preclinical support
Therapeutic targets	Iron chelators GPX4, SLC7A11, Nrf2, and ACSL4	Cytokines (IL-1β, TNF-α), iNOS, NLRP3, and NF-	Shared regulators include Nrf2, SIRT1, NF-κB, and redox enzymes	In CNS therapy, focusing on both arms could have a synergistic effect

Oxidative stress links ferroptosis and neuroinflammation tightly, and two ways are involved: one is that oxidative stress exacerbates neuroinflammation, and the other is that neuroinflammation results in oxidative stress. In neuroinflammation, activated microglia produce a tremendous amount of ROS and NO via activation of NADPH oxidase (NOX) and inducible nitric oxide synthase (iNOS) (Simpson and Oliver, 2020; Qu et al., 2022). These reactive molecules are involved in the promotion of membrane lipid peroxidation and are thought to promote iron-dependent ferroptosis of neurons. Additionally, inflammatory cytokines aggravate cellular vulnerability to ferroptosis by blocking the cystine/glutamate antiporter system Xc^–^, which is necessary for cystine uptake for the synthesis of glutathione (Ashraf et al., 2020). Lipid hydroperoxides cause ferroptosis when there is a deficit in glutathione, which decreases GPX4 activity (Dar et al., 2024). Importantly, maintenance of activation with M1 microglia further perpetuates this pathological pattern by perpetually releasing pro-inflammatory mediators and ROS, while M2 polarization can alleviate oxidative stress, restore redox homeostasis, and diminish ferroptosis-induced neural damage. These findings suggest that polarization of microglia is not only a reaction to ferroptosis but also plays an important role in disease development in acute and chronic CNS diseases.

Ferroptosis produces neuroinflammation-increasing DAMPs such as HMGB1, ATP, and oxidized lipids. Microglia and astrocytes express Toll-like receptors (TLRs) and nucleotide-binding oligomerization domain-like receptors (NLRs) that recognize this danger associated molecular patterns (DAMPs). These receptors, on activation, activate NF-κB signaling and inflammasome assembly, which results in an enhanced production of pro-inflammatory cytokines and chemokines (Gong et al., 2020). Iron from damaged neurons and pro-inflammatory fragments of ferroptotic cells are also inflammatory and activate microglia and astrocytes, leading to a further increase in neuroinflammation (Latunde-Dada, 2017; Ward et al., 2022). In addition to the above classical inflammatory pathways, there is growing evidence that non-coding RNAs, especially long non-coding RNAs (lncRNAs), play a vital role in the regulation of neuroinflammation and ferroptosis-related signaling. loncRNAs interact with various signaling pathways, such as NF-κB and NLRP3 signaling, and regulate inflammatory processes by regulating activation of immune cells, production of cytokines, oxidative stress and iron metabolism. In recent years, the role of dysregulated lncRNAs in remodeling the immune microenvironment and regulating inflammatory responses has been shown in glioma, implying that lncRNA-mediated immune regulation may also play a role in influencing the susceptibility to ferroptosis and a potential therapeutic target in CNS disorders (Zeng et al., 2022).

Chronic neurodegenerative diseases, like Parkinson’s and Alzheimer’s, have increased iron levels and inflammation in the substantia nigra and in the hippocampus after death (Snyder and Connor, 2009). Reactive oxygen species produced by iron overload drive misfolding, oligomerization, and aggregation of amyloid-β and α-Synuclein, two hall-mark proteins involved in Alzheimer’s and Parkinson’s diseases, respectively. These protein aggregates in turn further stimulate microglia and astrocytes, increasing the neuroinflammatory signaling and oxidative stress (Cahill et al., 2009). Recent studies show that ferroptosis plays a direct role in the pathological aggregation of proteins by causing excessive lipid peroxidation and the production of reactive aldehydes, such as 4-HNE, which can bind to proteins and alter their normal structure, functioning, and hallmark protein quality-control pathways. Oxidative modification of amyloid-β, α-synuclein and other proteins that aggregate increases their stability and neurotoxicity, and aggregated proteins worsen mitochondrial dysfunction, iron dyshomeostasis, and lipid peroxidation, which in turn reinforces ferroptotic signaling. This bidirectional process creates a vicious cycle of protein aggregation and ferroptosis-associated neuroinflammation and neuronal degeneration. The results highlight a novel mechanism by which ferroptosis can be a critical player in the progression of neurodegenerative diseases, and not just a downstream event.

One of the best examples of pathogenic interactions between the CNS and ischemic stroke and traumatic brain injury (TBI) (Kamel and Iadecola, 2012). During reperfusion, excessive oxidative stress and a disruption of blood-brain barrier integrity occur, allowing iron to enter the brain parenchyma and thus promoting ferroptosis during ischemic stroke. Neuronal injury can be further potentiated by the release of pro-inflammatory mediators and reactive oxygen species (ROS) by activated microglia and infiltrating peripheral immune cells (He et al., 2024). Experimental research has shown that compounds that inhibit lipid peroxidation, like ferrostatin-1, liproxstatin-1, and iron chelators such as deferoxamine, can reduce lipid peroxidation, neuronal death, and neuroinflammatory markers, making ferroptosis a potential therapeutic target (Cao et al., 2021).

These mechanisms, including mitochondrial dysfunction, glutathione depletion, iron dysregulation, and lipid peroxidation, are all involved in neuron death via ferroptosis, and sustained neuroinflammation is responsible for secondary brain injury in TBIs (Tang et al., 2020). Recent evidence also indicates that the composition of gut microbiota and gut–brain axis signaling are also altered after TBI and contribute to increased systemic inflammation and activation of the NLRP3 inflammasome. Immune signaling from gut dysregulation may further drive microglial activation, increased cytokine levels, and oxidative stress, thus further increasing the neuronal damage induced by ferroptosis. As a result, modulation of gut–brain axis has become a promising treatment option that can inhibit NLRP3-mediated neuroinflammation while promoting ferroptosis following traumatic brain injury (Zhao et al., 2025). The results confirmed that ferroptosis is linked to neuroinflammation, which indicates that these two pathophysiological mechanisms have interconnected mechanisms in acute and chronic CNS disorders (Cheng et al., 2021).

For instance, treatment with N-acetylcysteine could help reduce ferroptosis and inflammation, which involves increasing GPX4 levels, sustaining system Xc^–^ function, replenishing intracellular glutathione, and lowering iron-mediated oxidative stress (Han et al., 2024). Further, anti-inflammatory strategies to dampen the production of cytokines, prevent inflammasome activation, and lessen microglial hyperactivity could further inhibit oxidative damage and ferroptosis. Several naturally derived molecules have been shown to have dual anti-ferroptotic and anti-inflammatory properties, regulating oxidative stress, iron homeostasis, lipid peroxidation, and inflammatory pathways in recent studies. These are multi-target actions that suggest complementary strategies for blocking the pathogenic cross-influence between ferroptosis and neuroinflammation by natural compounds (Shaukat et al., 2025).

Although results are encouraging, there is still a long way to go before combination therapies could be made available to the clinic. The complicated and intricate molecular mechanisms involved in CNS disorders, the difficulty in getting therapeutic agents across the blood–brain barrier, uncertainty about the timing of intervention, and the absence of a ferroptosis-specific clinical marker are still major obstacles to successful clinical translation. In addition, over-inhibition of ferroptosis or inflammatory processes could disrupt physiological cellular homeostasis and immune defense, highlighting the importance of optimizing therapeutic approaches. Therefore, further research is needed to define disease-specific therapeutic windows, confirm valid disease markers and test combination therapies in carefully designed preclinical and clinical trials.

Ferroptosis and neuroinflammation are a complex pathway that is an underlying mechanism of both acute and chronic central nervous system diseases. All these molecules together create a vicious cycle that promotes further neuronal dysfunction and neurodegeneration (Woo et al., 2024). While there is significant experimental evidence in support of targeting ferroptosis and neuroinflammation as parallel therapeutic approaches, a number of knowledge gaps exist, including disease-specific molecular mechanisms, validation of biomarkers, long-term therapeutic safety, and clinical efficacy. Future studies should focus on combined multi-target strategies that modulate ferroptosis, oxidative stress, neuroinflammation, immune regulation, and also increase the delivery of the drugs to the brain via the blood–brain barrier. Better understanding of these interwoven pathways will help develop mechanism-based therapeutics that have greater translational value to patients suffering from CNS disorders.

## 4 Molecular mechanisms and signaling pathways

### 4.1 SIRT1: a molecular connection among intercommunication, neuroinflammation, and ferroptosis

A conserved member of the sirtuin family, SIRT1 is a nicotinamide adenine dinucleotide (NAD^+^)-dependent class III histone deacetylase that regulates cellular metabolism, oxidative stress response, aging, mitochondrial function, and inflammation (Zhang and Kraus, 2010). SIRT1 has been a subject of special interest as it is a protein that functions in several pathways, such as NRF2, NF-κB, AMPK, PI3K/Akt, MAPK, and p53 pathways, among others, involved in ferroptosis and neuroinflammation. SIRT1 does not only act on a single pathway to control a single aspect of cellular homeostasis, but rather it is an integrator of redox regulation, mitochondrial quality control, iron metabolism, lipid metabolism, and inflammatory signaling by binding to multiple downstream transcription factors and metabolic regulators. SIRT1’s regulation of multiple pathways suggests that it is not just a signaling molecule but rather a central molecular hub between ferroptosis and neuroinflammation. SIRT1 helps to maintain redox balance, mitochondrial function, lipid metabolism, and immune signaling; thereby, preventing oxidative damage and inflammatory injury to neurons (Xu et al., 2018). However, SIRT1 acts synergistically with other signaling pathways including NRF2, NF-κB, AMPK, and antioxidant signaling through KEAP1, creating a network of interconnected pathways controlling ferroptosis susceptibility and neuro-inflammation in CNS diseases.

#### 4.1.1 Defense against ferroptosis with SIRT1 and redox homeostasis

Ferroptosis, an iron-dependent regulated cell death, causes harmful lipid peroxide accumulation. Ferroptosis is crucial for breaking antioxidant defense mechanisms, specifically GPX4-mediated lipid hydroperoxide detoxification. SIRT1 regulates oxidative stress at various levels, affecting ferroptosis risk (Wang et al., 2023).

SIRT1 stimulates transcription factors such as FOXO3a, PGC-1α, and NRF2 by removing acetyl groups (Olmos et al., 2013). These factors produce more oxidative stress-protective genes. NRF2 controls cell redox balance. It regulates the transcription of genes like SLC7A11, GPX4, and HO-1, which affect iron and glutathione metabolism and ferroptosis sensitivity (Kerins and Ooi, 2018). SIRT1 indirectly boosts GPX4 and system Xc^–^ components (SLC7A11 and SLC3A2) expression by activating NRF2. This stops ferroptosis-critical lipid peroxidation (Tan et al., 2024).

In addition to transcriptional regulation, recent studies have also shown that ferroptosis is regulated at the post-transcriptional and translational levels. During oxidative stress, RNA-binding proteins, non-coding RNA, and translational regulatory mechanisms regulating the expression and activity of GPX4 and SLC7A11 are modulated, which ultimately results in protein stability and antioxidant capacity changes. These extra regulatory layers help identify how sensitive cells are to ferroptosis and could be a factor in the vulnerability of certain neurons in a disease. Moreover, GPX4 and SLC7A11 are emerging as potential targets for precision medicine strategies in CNS disorders, with promising results for promoting antioxidant protection and inhibiting ferroptotic cell death, thanks to the use of new nanomedicine-based therapeutic approaches (Zhong et al., 2025).

By signaling through PGC-1α, SIRT1 enhances mitochondrial biogenesis and function. This reduces mitochondrial ROS. SIRT1 improves ferritin and mitochondrial quality. This reduces the number of labile iron pools that start the Fenton reaction, which produces lipid peroxides. SIRT1 strongly inhibits ferroptotic cell death (Latunde-Dada, 2017).

#### 4.1.2 SIRT1 and neuroinflammation: signaling regulation

Neurodegenerative and acute central nervous system disorders like Alzheimer’s, Parkinson’s, MS, and TBI are characterized by neuroinflammation (Stephenson et al., 2018). Pro-inflammatory cytokines (such as IL-1β, TNF-α, and IL-6) and reactive oxygen/nitrogen species (ROS/RNS) are released when microglia and astrocytes are activated. Iron dysregulation and oxidative stress, which are connected to ferroptosis pathways, are exacerbated by chronic neuroinflammation, leading to neuronal dysfunction and death (David et al., 2022).

SIRT1 regulates inflammatory transcription factors, such as NF-κB, to reduce neuroinflammation. SIRT1 removes acetyl groups from the RelA/p65 component of NF-κB (Singh and Ubaid, 2020; Song et al., 2022). Lower transcriptional activity decreases pro-inflammatory cytokines and chemokines. Blocking NF-κB is crucial for preventing persistent glial activation and maintaining central nervous system immunological homeostasis (Shih et al., 2015).

G protein-coupled receptors (GPCRs) have also been recently discovered as key upstream regulators of neuroinflammatory signaling. The activation of GPCRs activates intracellular signaling pathways that link extracellular inflammatory stimuli with intracellular signaling pathways involving NF-κB, MAPK, PI3K/Akt and cyclic AMP-dependent pathways, which in turn modulate microglial activation, cytokine production, and responses to oxidative stress. Recent studies also show that GPCR signaling regulates intracellular redox homeostasis, lipid metabolism, and production of inflammatory mediators, suggesting it also interacts with ferroptosis-related pathways. Overall, the results indicate that GPCRs are potential targets in the treatment of CNS diseases because they can simultaneously regulate the neuroinflammatory response and ferroptotic susceptibility (Guo et al., 2025).

SIRT1 interacts with other inflammatory processes. The NLRP3 inflammasome is involved in neuroinflammatory and ferroptotic processes. SIRT1 deacetylates and stabilizes FOXO3a to inhibit NLRP3. This promotes genes that combat inflammation and free radicals, inhibiting inflammasome-induced pyroptosis and tissue damage (Zhu et al., 2024).

There are multiple complementary pathways by which evidence has suggested that SIRT1 may inhibit NLRP3 inflammasome activation, but not a single pathway. The deacetylation of the NF-kB p65 subunit by SIRT1 has been shown to decrease transcription of the components of the NLRP3 inflammasome and pro-inflammatory cytokines such as pro-IL-1β and pro-IL-18. Moreover, SIRT1 helps maintain mitochondrial integrity and stimulates mitochondrial biogenesis by activating PGC-1α, thereby lowering the release of mitochondrial reactive oxygen species, which are significant factors that trigger the assembly of the NLRP3 inflammasome. The activation of NRF2 also increases cellular antioxidant defenses, thereby reducing oxidative stress-induced inflammasome activation, further dampening inflammasome activation. In this context, SIRT1 has a protective role as a prominent coordinator of CNS homeostasis and functions simultaneously to inhibit both ferroptosis and neuroinflammation by reducing oxidative stress, maturation of inflammatory cytokines, and excessive activation of the inflammasome.6.1.3. SIRT1 links ferroptosis with inflammation.

SIRT1 stops lipid peroxidation, which causes ferroptosis, and lowers pro-inflammatory signaling, a neuroinflammation symptom, regulating these two processes (Zhang et al., 2025). Inflammatory chemicals like IL-1β or TNF-α can lead to CNS disorders by causing ROS, glutathione depletion, and iron release. All of these increase ferroptosis-prone neurons. Ferroptosis-related reactive oxygen species and lipid peroxidation by-products like 4-HNE activate microglia and astrocytes, worsening neuroinflammation (Liu et al., 2024). This negative cycle can be broken by SIRT1-mediated feedback loop suppression (Xie et al., 2019).

Pharmaceutical activation of SIRT1 (e.g., resveratrol, SRT2104) can decrease neuronal loss in models of stroke, Alzheimer’s, and Parkinson’s disease by suppressing ferroptotic and inflammatory pathways (Chang et al., 2024). Stopping SIRT1 promotes glial activation and oxidative stress sensitivity in neurons (Khan et al., 2012).

#### 4.1.4 Clinical implications

Ferroptosis and neuroinflammation may be treated by targeting SIRT1 (Jiao and Gong, 2020). We need to understand SIRT1’s effects in different circumstances to understand them fully. SIRT1 may have harmful pro-survival effects in tumor microenvironments or autoimmune diseases (Bustos et al., 2020). Thus, SIRT1 therapy modification must be tailored to each disease’s pathophysiological conditions.

SIRT1’s molecular switches in ferroptosis and inflammation should be identified in future research. This includes its interactions with other sirtuins (SIRT3), redox sensors (KEAP1), and metabolic regulators (AMPK) (Chen M. et al., 2024). SIRT1’s action in neurons versus glia may reveal focused therapeutic insights. Brain-penetrant SIRT1 activators with improved pharmacokinetics may enable innovative CNS treatments for ferroptotic and inflammatory disorders (Hugo et al., 2024). The molecular linkages between ferroptosis and neuroinflammation require SIRT1 to modulate oxidative stress, iron metabolism, and immune signaling (Liu et al., 2025). Its varied activities improve neuroprotection and make SIRT1 a promising biological target for CNS diseases with ferroptotic and inflammatory damage (Jiao and Gong, 2020).

As the connection of SIRT1 to ferroptosis and neuroinflammation has been increasingly recognized, there has been a lot of interest in the development of SIRT1-targeting therapeutics. A number of pharmacological SIRT1 activators, including natural molecules like resveratrol and synthetic molecules, have been found to exert neuroprotective effects in experimental models through mechanisms involving the decrease of oxidative stress, inhibition of inflammatory signaling, and maintenance of mitochondrial function. There are, however, a number of important problems to be addressed in translation. Due to its role in various physiological processes, many SIRT1 activators are currently available that have limited specificity, low bioavailability and varying degrees of blood–brain barrier penetration, and prolonged or excessive activation of SIRT1 can result in off-target metabolic effects. The restraints underscore the necessity of creating more specific SIRT1 modulators and improved drug delivery systems. Moreover, future therapeutic approaches will likely involve the simultaneous modulation of complementary signaling pathways (such as NRF2, NF-κB, AMPK, and GPCR-mediated signaling) to generate more comprehensive control over oxidative stress, ferroptosis, and neuroinflammation. These poly-targeted therapies, along with disease-specific markers and precision medicine therapies, could offer improved clinical treatments for central nervous system diseases.

### 4.2 Nrf2: an important control point between ferroptosis and neuroinflammation

A basic leucine zipper (bZIP) transcription factor called nuclear factor erythroid 2–related factor 2 (Nrf2) is essential for maintaining cell redox homeostasis (He et al., 2020). It controls roughly 200 cytoprotective genes involved in detoxification, antioxidant defense, mitochondrial function, iron metabolism, and inflammation (Brigelius-Flohé and Flohé, 2011). The CNS regulates oxidative stress, neuroinflammation, and ferroptosis through Nrf2. Nrf2 signaling dysregulation is linked to CNS diseases such as Alzheimer’s, Parkinson’s, stroke, TBI, and multiple sclerosis (Abdalkader et al., 2018; Song and Long, 2020).

#### 4.2.1 Nrf2 and ferroptosis: iron and antioxidant control

Ferroptosis is a controlled necrosis that accumulates lipid peroxides and requires iron. GPX4 converts lipid hydroperoxides into harmless alcohol derivatives. GSH is needed for this. This ferroptosis prevention technique is significant. Nrf2 controls GPX4 and GSH production. It is a major barrier to ferroptotic cell death before it (Wang et al., 2021).

Nrf2 increases the expression of SLC7A11, a component of the Xc^–^ system that transports cystine and glutamate (Jyotsana et al., 2022). This helps cells absorb cystine. Cysteine to cysteine is the first step in glutathione production. GPX4 works better and lowers lipid peroxidation when Nrf2 makes GSH more available. Nrf2 increases GCLM and GCLC transcription. These two components aid and affect glutamate-cysteine ligase. The first glutathione-making enzyme (Solis et al., 2002; Iles and Liu, 2005).

Ferroptosis and antioxidant defenses depend on Nrf2’s regulation of intracellular iron levels. It changes iron storage and release, gene activation, and deactivation (Liu et al., 2022). Ferritin heavy chain 1 (FTH1), light chain (FTL), and ferroportin (FPN1/SLC40A1) genes are examples (Luscieti, 2016). Proteins reduce the labile iron pool, preventing the Fenton reaction from producing hydroxyl radicals and lipid peroxidation. Nrf2 reduces TfR1 and DMT1 to block iron entry (Kontoghiorghes and Kontoghiorghe, 2020). Nrf2 regulates heme oxygenase-1 (HO-1), which transforms heme into biliverdin, carbon monoxide, and free iron (Consoli et al., 2021). At normal levels, HO-1 protects cells, whereas overactivation releases iron and causes ferroptosis. Nrf2 modulates HO-1, emphasizing the importance of a calibrated response (Yuan et al., 2024).

#### 4.2.2 Nrf2 and neuroinflammation: anti-inflammation signaling pathways

Neuroinflammation, which includes glial cell activation and the release of pro-inflammatory cytokines, chemokines, and reactive oxygen/nitrogen species, is a hallmark of many CNS disorders (Thompson and Tsirka, 2017). Long-term inflammation increases oxidative stress and neuron loss. Nrf2 inhibits pro-inflammatory gene expression and speeds inflammation repair (Shetty et al., 2017).

Nrf2 primarily combats inflammation by blocking NF-κB signaling. NF-κB is a vital transcriptional regulator of inflammation. Nrf2 inhibits NF-κB via competing for transcriptional co-activators like CBP/p300, increasing antioxidant enzyme production, and stopping upstream signaling kinases like IKK. This technique prevents NF-κB from producing cytokines such as IL-1β, TNF-α, and IL-6 (Saha et al., 2020).

The NLRP3 inflammasome, which generates pro-inflammatory cytokines like IL-1β and IL-18, is inhibited by Nrf2 activation (Chen et al., 2019). Nrf2 reduces mitochondrial ROS and mitochondrial dysfunction, which activate NLRP3, to prevent inflammasome activation. Pyroptosis, an inflammatory cell death comparable to ferroptosis, is prevented by Nrf2 (Rusetskaya et al., 2023). Turning on Nrf2 switches microglia from pro-inflammatory M1 to neuroprotective M2. M2 microglia release growth factors and anti-inflammatory cytokines to protect neurons and heal tissues. The phenotypic change restores central nervous system balance and reduces long-term neuroinflammation (Guo et al., 2022).

#### 4.2.3 Ferroptosis-induced inflammation and Nrf2 function

Ferroptosis and neuroinflammation interact synergistically. DAMPs, lipid peroxidation by-products such as 4-hydroxynonenal and malondialdehyde, and ROS from ferroptotic cell death activate microglia and astrocytes with pro-inflammatory signals (Chen R. et al., 2024). Worsening oxidative stress and inflammation perpetuate the cycle. This cycle uses Nrf2 as a molecular “inhibitor.” Ferroptosis and inflammatory signals are reduced, disrupting the pathogenic amplification loop between oxidative damage and immunological activation (Park et al., 2023). Pharmacological Nrf2 activators such as sulforaphane, bardoxolone methyl, and dimethyl fumarate can prevent neuroinflammatory damage and ferroptotic neuronal loss in ischemic stroke, spinal cord injury, and dementia, according to experimental models (Scuderi et al., 2020).

#### 4.2.4 Clinical implications

Nrf2 regulates ferroptosis and neuroinflammation, making it a promising central nervous system disease treatment target. Clinical and preclinical trials are underway for many Nrf2 activators (Song and Long, 2020). Dimethyl fumarate (DMF), an FDA-approved multiple sclerosis treatment, shows how Nrf2 activation in the CNS can be beneficial. DMF lowers inflammation and boosts free radical defenses. This improves neuron function and lifespan (Zhao et al., 2015).

Long-term and unregulated Nrf2 activation can impair the immune system and iron metabolism. Thus, future approaches must regulate Nrf2 activity according to tissue, cell type, and circumstance (Kerins and Ooi, 2018). Nanoparticle-based delivery technologies and gene-editing may focus Nrf2’s defensive activity and reduce body-wide impacts (Yin et al., 2024).

Nrf2, a transcription factor, controls oxidative stress, ferroptosis, and neuroinflammation. Nrf2 regulates iron metabolism, redox balance, glutathione production, and inflammatory signaling to maintain central nervous system equilibrium (Song and Long, 2020). Therapeutic techniques that increase Nrf2 activity may alleviate ferroptotic and inflammatory neurodegeneration in several central nervous system diseases (Suzen et al., 2022).

### 4.3 NF-κB: an important link between communication, neuroinflammation, and ferroptosis

In the central nervous system, the NF-κB signaling pathway is essential for controlling inflammation, cell survival, immunological response, and stress adaptation. The transcription factors RelA (p65), RelB, c-Rel, NF-κB1 (p50/p105), and NF-κB2 (p52/p100) comprise the NF-κB family (Grilli and Memo, 1999). These proteins can form a variety of hetero- and homodimers. IκB proteins keep inactive NF-κB dimers in the cytoplasm. When cytokines, ROS, PAMPs, or DAMPs are present, the IKK complex phosphorylates and breaks down IκB. This enables the entry of NF-κB into the nucleus and starts the transcription of particular genes (Senftleben and Karin, 2002; Scheidereit, 2006).

It is well recognized that NF-κB plays a part in inflammation by encouraging the synthesis of genes that code for adhesion molecules, cytokines, chemokines, and enzymes like COX-2 and Inos (Surh et al., 2001). Recent studies have shown that it is altering the way it regulates ferroptosis, which is characterized by lipid peroxide accumulation and is a controlled, iron-dependent cell death that differs from apoptosis (Cheng et al., 2021; Wang et al., 2023).

#### 4.3.1 NF-κB in neuroinflammation: primary regulator of innate immune activation

NF-κB significantly influences neuroinflammation. It permits immune cells from outside the central nervous system to enter and stimulates indigenous glial cells such as astrocytes and microglia (Singh et al., 2022). The NF-κB pathway in microglia begins with the activation of TLRs, TNFRs, and IL-1Rs. This activates pro-inflammatory mediator transcription. Among the chemicals that poison the brain and draw in more immune cells are IL-1β, TNF-α, IL-6, MCP-1, and CCL2 (Zehra et al., 2025).

NF-κB activation also increases the production of nitric oxide (NO) and superoxide by inducible iNOS and NADPH oxidase (Brady et al., 1997). This exacerbates lipid peroxidation and oxidative stress. By aiding in the disintegration of organelles and neuronal membranes, these reactive oxygen species increase the likelihood of ferroptotic cell death in the environment (Wang et al., 2023).

In the event of a central nervous system injury, astrocytes activate NF-κB. Consequently, the blood-brain barrier (BBB) becomes thinner and produces pro-inflammatory cytokines. Persistent NF-κB activation in astrocytes promotes the establishment of glial scars and impedes tissue healing in chronic conditions (Archie et al., 2021).

#### 4.3.2 Are NF-κB and ferroptosis friends or foes?

Although its function in ferroptosis is complicated and context-dependent, NF-κB is a known survival factor. Ferroptosis can be facilitated by NF-κB, a pro-oxidant. NF-κB signaling activates inflammatory mediators such as IL-1β, TNF-α, and LPS, which results in the generation of ROS and the build-up of iron (Kurmangaliyeva et al., 2024). These are the main stages of ferroptotic cell death. Lipid peroxidation, a defining feature of ferroptosis, may result from NF-κB activation speeding up the synthesis of ALOX15, an enzyme that facilitates PUFA peroxidation (Zheng et al., 2024).

Under specific circumstances, NF-κB prevents the synthesis of SLC7A11, an essential part of the cystine/glutamate antiporter (system Xc^–^). GSH synthesis and cell cystine intake are decreased as a result. The GPX4 enzyme stops working. Because GPX4 requires glutathione and breaks down lipid peroxides, ferroptosis is more likely (Chen et al., 2025). By triggering oxidative stress-fighting enzymes or apoptosis-preventing genes (like Bcl-2 and IAPs), NF-κB-dependent pathways can inhibit ferroptosis. Ferroptosis may be avoided by storing iron and lowering free iron levels through NF-κB-mediated ferritin increases. Therefore, depending on the cell and environment, NF-κB can either facilitate or hinder ferroptosis (Sze et al., 2022).

#### 4.3.3 NF-κB-mediated feedback loop: connecting inflammation to ferroptosis

In the destructive cycle brought on by ferroptosis and neuroinflammation, NF-κB plays a critical role. By generating DAMPs such as HMGB1, ATP, 4-HNE, and MDA, ferroptosis causes neuroinflammation (Tjepkema, 2024). These DAMPs activate NF-κB in glial cells through pattern recognition receptors (PRRs). This exacerbates ferroptosis in neighboring neurons by raising pro-inflammatory mediators and reactive oxygen species (Kaur et al., 2023).

Particularly hazardous is this positive feedback loop in cases of chronic CNS disorders. Amyloid-β accumulation in Alzheimer’s disease triggers microglial NF-κB, which results in ferroptotic cell death from lipid peroxidation and iron dysregulation (Firdous et al., 2024). NF-κB activation in endothelial and glial cells accelerates neuronal death in ischemic stroke, causing increased inflammation and ferroptosis in the penumbra (Xu et al., 2021).

A key biochemical connection between ferroptosis and neuroinflammation of the central nervous system is NF-κB (Gao et al., 2023). Neuronal sensitivity to ferroptosis is increased by increased pro-inflammatory gene activity, ROS generation, and altered iron and lipid consumption by NF-κB. Ferroptosis-related DAMPs increase NF-κB signaling, which starts a self-replicating neurotoxic cycle. Developing effective treatments for CNS disorders requires an understanding of NF-κB processes and effects (Deng et al., 2023).

### 4.4 PTGS2/COX-2: an important link between ferroptosis and neuroinflammation in the pathophysiology of the central nervous system

Cyclooxygenase-2 (COX-2) or prostaglandin-endoperoxide synthase 2 (PTGS2) is an enzyme that can be produced in response to specific circumstances (Jaén et al., 2018). It accelerates the synthesis of prostanoids from arachidonic acid, including prostaglandin E2 (PGE2). The body ordinarily contains low amounts of COX-2, but when oxidative stress or inflammation occurs, the amount of COX-2 increases significantly (Minghetti, 2004). In the central nervous system (CNS), COX-2 has a variety of roles in ferroptosis and neuroinflammation. It serves as a molecular link between the two mechanisms. The expression of genes is meticulously regulated by transcription factors such as AP-1, CREB, and NF-κB. These factors are activated by oxidative damage and inflammatory stimuli (Müller et al., 1997).

Recently, COX-2 has become a prominent downstream effector and inflammatory marker. It could potentially indicate ferroptosis, a regulated, iron-dependent form of cell death characterized by lipid peroxidation (Xu et al., 2022). A number of CNS diseases, such as Alzheimer’s disease (AD), Parkinson’s disease (PD), stroke, TBI, and multiple sclerosis (MS), are greatly influenced by the convergence of inflammatory and ferroptotic signals via COX-2 in glial and neuronal cells (Ou et al., 2022).

#### 4.4.1 PTGS2/COX-2 in neuroinflammation: catalyst and enhancer

Pro-inflammatory cytokines such as IL-1β and TNF-α rapidly activate COX-2. Additionally, PAMPs, including lipopolysaccharide (LPS), serve as another example. In the inflamed central nervous system, activated microglia, astrocytes, and neurons exhibit high levels of expression (Rodríguez-Gómez et al., 2020). COX-2 converts arachidonic acid into prostaglandin H2 (PGH2), which is subsequently transformed into various prostanoids. Among these, PGE2 stands out as a significant pro-inflammatory lipid mediator (Kulesza et al., 2023).

PGE2 interacts with its receptors (EP1–EP4) to modify the immune system, facilitate the crossing of the blood-brain barrier, influence cytokine production, and affect synaptic transmission. PGE2 increases pro-inflammatory (M1-like) microglia and astrocytes, resulting in increased IL-6, TNF-α, and iNOS levels (Sanjay et al., 2022).

The strong positive feedback loop between NF-κB activation and COX-2 expression is crucial for long-lasting neuroinflammation (Johansson et al., 2015). NF-κB upregulates PTGS2 transcription, and COX-2 signaling produces prostaglandins that activate it. The inflammatory response continues, worsening neurodegeneration (Tyagi et al., 2020).

#### 4.4.2 PTGS2 and COX-2 serve as both biomarkers and mediators of ferroptosis

In addition to its pro-inflammatory characteristics, COX-2 is recognized as a significant downstream indicator of ferroptosis, especially concerning neuronal injury (Mohan et al., 2024). The accumulation of lipid hydroperoxides brought on by iron, primarily from polyunsaturated fatty acids (PUFAs) in phospholipid membranes, is a characteristic of ferroptosis (Mortensen et al., 2023). When antioxidant defenses, especially GSH and GPX4, are overpowered, oxidative damage results, leading to cell death (Pei et al., 2023).

PTGS2/COX-2 is transcriptionally increased during ferroptosis, independent of conventional inflammatory stimuli (Wu et al., 2024). This rise is thought to be a last stage in the ferroptotic cascade and is brought on by oxidative stress inside the cell. COX-2-produced prostanoids promote ferroptotic signaling by facilitating membrane lipid oxidation and cellular death. Ferroptosis can be reliably detected in both living organisms and in the laboratory by high levels of COX-2 expression. This is used by scientists to assess the effectiveness of ferroptosis in experimental models (Costa et al., 2023; Deng et al., 2023).

By-products of COX-2 enzymatic activity, such as prostaglandin-endoperoxides and thromboxanes, can exacerbate lipid peroxidation, which in turn can cause ferroptosis (Tada and Suzuki, 2016). Therefore, COX-2 may have a dual role in ferroptotic cell death, both as an active mediator and as a passive participant, particularly in the central nervous system that is oxidatively stressed or inflamed (Tyurina et al., 2019).

#### 4.4.3 Therapeutic considerations: inhibiting COX-2 in central nervous system disorders

COX-2 may be utilized to treat illnesses of the central nervous system that are linked to the oxidative and immunological systems because it is implicated in ferroptosis and neuroinflammation (Cheng et al., 2021). Selective COX-2 inhibitors, such as celecoxib and rofecoxib, decrease prostaglandin production, inflammation, and neuronal damage in animal models of stroke, Alzheimer’s disease, and spinal cord injury (Minghetti, 2004).

By preventing lipid peroxidation and oxidative damage, the inhibition of COX-2 may also enhance the resistance of cells to ferroptosis. Inhibitors that serve dual functions, regulating COX-2 alongside upstream ferroptosis regulators such as GPX4 and SLC7A11, or those administered with a focus on the central nervous system, are advised due to the association of prolonged systemic COX-2 inhibition with cardiovascular risks (Mallick et al., 2025). It takes integrated therapy approaches that alter COX-2 and anti-inflammatory/antioxidant pathways, including Nrf2 activation or SIRT1 stimulation, to stop inflammation-induced ferroptosis (Wang et al., 2025).

Ferroptosis and neuroinflammation exhibit a reciprocal relationship that relies on PTGS2/COX-2. The regulation of lipid peroxidation and inflammation induced by prostaglandins is managed by COX-2 (McAdam et al., 2000). This links oxidative neuronal death in CNS disorders to immunological stimulation. Its role as a neurodegenerative molecular hub is demonstrated by its elevation in response to ferroptotic and inflammatory signals (Ward et al., 2022). Concentrating on COX-2 and its signaling pathways may help mitigate ferroptosis and brain inflammation (Xu et al., 2022).

### 4.5 iNOS/NO●: a mediator in intercellular communication, neuroinflammation, and ferroptosis in the central nervous system

Induced iNOS, also known as NOS2, is responsible for the production of NO●, RNS (Thomas et al., 2015). Neurotransmission, immunological response, vascular tone, and oxidative stress are all impacted by NO●. Under normal conditions, iNOS is rarely detectable, in contrast to constitutively produced isoforms such as nNOS and eNOS (Iova et al., 2023). However, iNOS levels are markedly elevated by pro-inflammatory stimuli such as lipopolysaccharide, TNF-α, IL-1β, and IFN-γ (Cho et al., 2008).

NO● generated from iNOS mediates and intensifies immunological responses in neuroinflammation (Liu et al., 2002; Akbar et al., 2016). According to recent research, ferroptosis—a controlled cell death brought on by iron-dependent lipid peroxidation—is impacted by iNOS/NO● signaling (Qu et al., 2022). The inflammatory and ferroptotic signaling of iNOS emphasizes its function in the immunological response and redox-mediated cell death of disorders of the central nervous system (CNS) (Ma et al., 2025).

#### 4.5.1 iNOS/NO● in neuroinflammation: stimulation and enhancement

**FIGURE 4:**
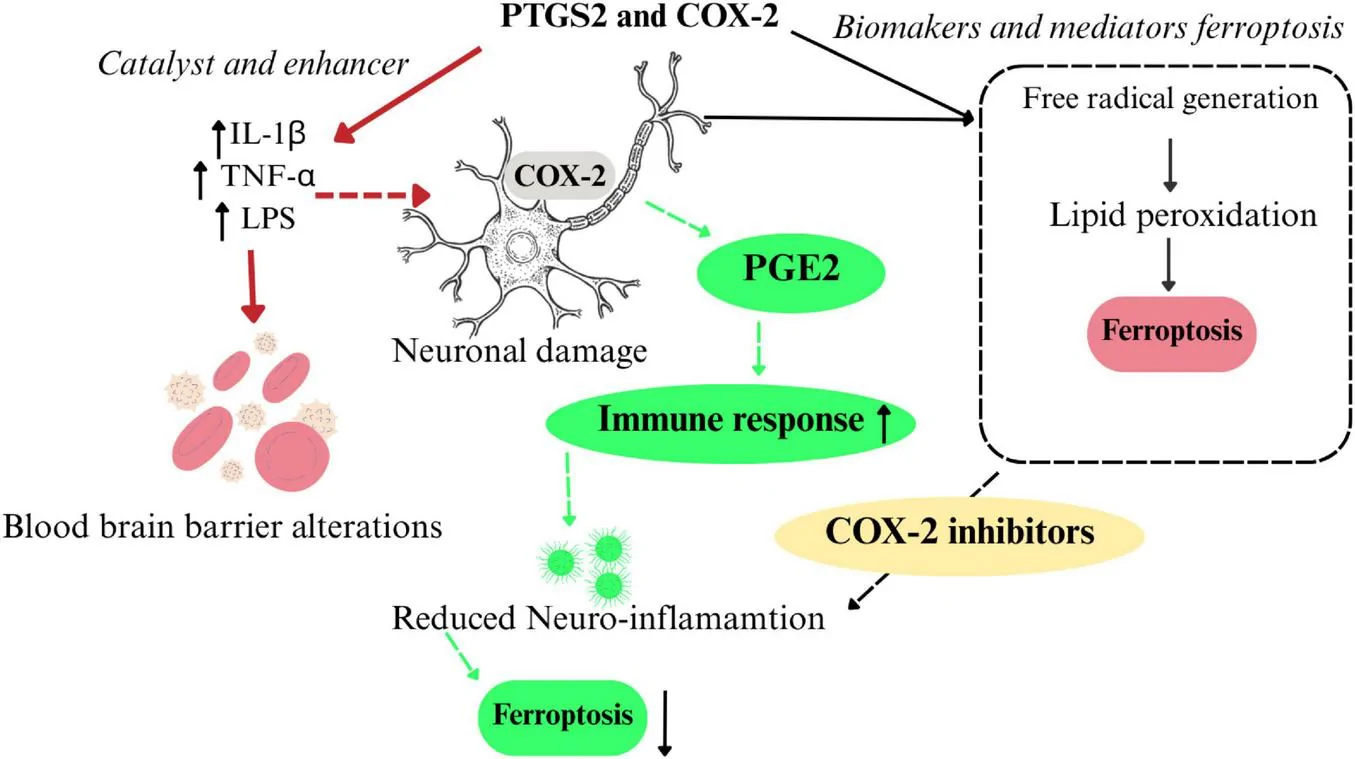
A schematic illustration of PTGS2/COX-2 promoting neuroinflammation and ferroptosis, and it also emphasizes the possibility of COX-2 suppression as a treatment approach.

iNOS rises in response to inflammatory stimuli that activate astrocytes and microglia, prolonging the generation of NO● (Figure 4). In contrast to nNOS and eNOS, which carefully regulate the production of minute amounts of NO● (Aktan, 2004).

Despite variations in cell calcium levels, calcium/calmodulin regulates iNOS transcription and generates a large amount of NO●. Overexpression of iNOS is caused by the transcription factors NF-κB, STAT1, and IRF1 (Michalak and Michalak, 2025). These factors are activated by PAMPs and cytokines. NO, which is produced by iNOS, has two purposes in the central nervous system (Szelényi, 2001). At regular dosages, it acts as a vasodilator and neurotransmitter. Damage-causing peroxynitrite (ONOO^–^), a reactive chemical, is produced during neuroinflammation by an excess of nitric oxide (NO●) and superoxide anion (O_2_^●–^) (Di Giulio and Meyer, 2008). Peroxynitrite can damage DNA, block mitochondrial enzymes, nitrate protein tyrosine residues, and kill cells. By oxidizing membrane phospholipids, ONOO^–^ connects ferroptosis pathways to RNS produced from iNOS (Abalenikhina et al., 2020).

#### 4.5.2 iNOS/NO● and ferroptosis

The role of NO● in ferroptosis is situational and intricate. Lipid peroxidation, a symptom of ferroptosis, can result from nitrosative stress caused by NO● from iNOS, which can also reduce antioxidant levels such as GSH and make GPX4 less active (Gao et al., 2021; Tan et al., 2023). Lipids and proteins are directly oxidized by NO and its by-products, including peroxynitrite (ONOO^–^), which maintains redox equilibrium. Cysteine residues on GPX4 or system Xc^–^ (SLC7A11) can be S-nitrosylated by NO●. Ferroptosis increases as a result of this inhibition of glutathione synthesis and cystine absorption (Wang et al., 2024).

However, some studies indicate that ferroptosis may be suppressed by small amounts of NO●. NO can halt lipid radical chain reactions and peroxidation by nitrosating lipid peroxyl radicals. In inflammatory environments with high levels of iNOS expression and NO● generation, its antioxidant qualities are typically undetectable (Rubbo et al., 1996; Bloodsworth et al., 2000).

By interacting with labile iron pools, nitric oxide increases Fenton chemistry and produces extremely reactive hydroxyl radicals, which intensify lipid peroxidation in inflammatory central nervous system illnesses (Adibhatla and Hatcher, 2010). Furthermore, ferroptosis-related ROS generation and mitochondrial dysfunction can be exacerbated by iNOS-mediated NO, which can interfere with mitochondrial electron transport (Zhang et al., 2023).

#### 4.5.3 Reciprocal relationships: ferroptosis-driven inflammation and inflammation-induced ferroptosis

The relationship between ferroptosis and iNOS/NO● signaling is essential in CNS disorders involving oxidative and immunological damage (Yan et al., 2021). Increased iNOS expression in response to neuroinflammatory stimuli results in a weaker cellular antioxidant defense mechanism, increased NO● production, and increased ROS/RNS generation (Aquilano et al., 2008). This increases the likelihood of ferroptosis-induced neuronal death. Damage-associated molecular patterns (DAMPs) and lipid peroxidation by-products (such as 4-HNE and MDA) produced by ferroptotic cell death stimulate glial cells and increase the synthesis of iNOS, creating a negative feedback loop (Wang et al., 2026).

Additionally, NO● can help ferritin break down and iron-sulfur clusters release iron. This increases the availability of iron that may go through redox processes, which is a crucial component in ferroptosis. This interaction places iNOS/NO● at a critical juncture between redox-mediated lipid catabolism and pro-inflammatory signaling (Lee and Roh, 2023).

#### 4.5.4 Clinical implications

Neuroinflammatory and ferroptotic CNS illnesses may be cured by focusing on iNOS and NO● signaling. iNOS inhibitors such as aminoguanidine and 1400W decrease neuroinflammation, oxidative damage, and neuronal degeneration in animal models of stroke, traumatic brain injury, and neurodegeneration (David et al., 2022; Ji et al., 2024).

By decreasing inflammation, NO●/ONOO^–^ production, and antioxidant defenses, combinatorial approaches that decrease iNOS and increase Nrf2 or SIRT1 activity can enhance neuro-protection (Song et al., 2022). Because NO● plays physiological roles in vascular control and neurotransmission, therapeutic methods need to be carefully thought out and carried out to prevent systemic consequences (Sándor, 1999).

Ferroptosis and neuroinflammation of the central nervous system depend on the iNOS/NO● axis (Qu et al., 2022). Induced iNOS produces NO●, which speeds up lipid peroxidation, interferes with antioxidant mechanisms, and increases oxidative and nitrosative stress (Kurutas, 2015). It results in ferroptotic cell death and inflammation. Keep in mind that NO● influences immunity and cell fate. This aids in the customization of neurodegenerative therapies brought on by ferroptosis and inflammation (Li et al., 2024).

### 4.6 Nutritional modulation of ferroptosis: implications for neuroprotection and healthy aging

Ferroptosis is a very sensitively regulated process with nutritional and metabolic signals, where its performance is directly regulated by iron supply, lipid peroxidation processes, and cellular antioxidant levels. Ferroptic vulnerability is moderated by diets, antioxidant agents, and minor bioactive molecules by regulating fundamental controlling mechanisms, which comprise GPX4, glutathione metabolism, cystine/glutamate antiporter system Xc^–^, and iron homeostasis.

Selenium is a crucial micronutrient which is required in the production and enzymatic activity of GPX4, the key enzyme that breaks phospholipid hydroperoxides. Deficiency in selenium leads to a reduction in GPX4 activity, an increase in lipid peroxidation, and a predisposition of neurons to ferroptosis, and adequate selenium intake of the nutrient increases’ antioxidant defenses and neuronal survival. Lipid-soluble antioxidant vitamin E is a direct scavenger of lipid peroxyl radicals and a potent endogenous ferroptotic inhibitor, especially in polyunsaturated fatty acid neuronal membranes.

Indirectly, polyphenolic compounds [resveratrol, curcumin, and epigallocatechin gallate (EGCG)] reduce ferroptosis through the stimulation of the Nrf2 and SIRT1 pathways. These signaling pathways enhance glutathione production, enhance mitochondrial redox homeostasis, and control iron-handling proteins, which lessen ferroptosis-related oxidative injury. The amino acids and glutathione precursors containing sulfur, such as cysteine and N-acetylcysteine, help to sustain system Xc^–^ activity and maintain intracellular glutathione levels, which sustain GPX4 activity.

In addition, small bioactive molecules like ferrostatin-1 and liproxstatin-1 are direct inhibitors of lipid peroxidation, and they have shown strong neuroprotectant properties against stroke, traumatic brain damage, and neurodegeneration in experimental models. Pharmacological chelators of iron, such as deferoxamine, decrease the pool of labile iron and limit Fenton chemistry, which further reduces ferroptotic neuronal death.

All these findings point to the direction that ferroptotic nutritional and dietary modulation is an effective non-invasive approach to healthy aging and neuroprotection. Diet-based interventions coupled with pharmacological interventions that suppress ferroptosis can be synergistically beneficial in the prevention or delay of ferroptosis-driven neurodegenerative mechanisms.

Ferroptosis is a highly controlled form of cell death that is significantly sensitive to nutritional factors, redox homeostasis, and iron levels, which makes it a central point of dietary and metabolic modulation. Recent engineering-related studies emphasize the combination of nutritional redox with systems-level regulation of oxidative stress and iron metabolism, with diet being a determinable factor of cellular susceptibility to oxidative damage and of the controlled cellular responses to oxidative stress, such as ferroptosis (Zehra et al., 2025; Zehravi et al., 2022; Zeng et al., 2022).

Selenium is a central micronutrient that is necessary in biosynthesis and catalysis of GPX4. Research studies on redox-active micronutrients have identified the role of selenium in maintaining lipid peroxide detoxification and oxidative membrane damage countermeasures, especially in highly metabolic tissues like those in the central nervous system (Zhang et al., 2014; Zhang and Kraus, 2010). Selenium deficiency compromises the work of GPX4, thus enhancing lipid peroxidation and exposing the neurons to ferroptosis. On the other hand, adequate intake of selenium strengthens endogenous antioxidant defenses and sustains survival of neurons. At the same time, the lipid-soluble antioxidant vitamin E has been shown to directly neutralize lipid-peroxyl radicals, and has been recognized in the engineering and biomaterials context as a naturally occurring ferroptosis-inhibitor, especially in polyunsaturated fatty-acid-rich neuronal membranes (Zhang et al., 2023; Zhang et al., 2025).

In addition to the traditional micronutrients, diet polyphenolic compounds, including resveratrol, curcumin, and epigallocatechin -gallic acid (EGCG), have a modulatory effect on ferroptosis through an indirect stimulation of Nrf2 -and SIRT1-dependent pathways. The latest articles in the engineering field highlight that these signaling pathways are key regulatory nodes that combine metabolic activity, mitochondrial bioenergetics, antioxidant gene expression, and iron-homeostasis signaling, and hence, suppressing ferroptotic-related oxidative stress on a systems level (Zhao et al., 2015; Zhao et al., 2023). Activation of Nrf2 enhances glutathione biosynthesis and GPX4 expression, while SIRT1 improves mitochondrial redox homeostasis and suppresses inflammatory-oxidative feedback loops.

Amino acids and glutathione precursors that contain sulfur, e.g., cysteine and N-acetylcysteine, also regulate ferroptosis resistance by keeping the Xc^–^ transporter active and protecting intracellular glutathione stores. Metabolic studies that include engineering studies lay stress on the importance of cystine exchange systems to glutamate in maintaining cellular redox homeostasis during oxidative stress and inflammatory stress situations (Zhao et al., 2025), thus maintaining the activity of GPX4 and preventing lipid peroxidation.

Taken together, the evidence confirms the hypothesis that ferroptotic nutritional and dietary regulation is an effective, non-invasive neuroprotective method and healthy aging approach. Engineering and systems-medicine perspective: On the one hand, diet-based interventions, with the addition of pharmacological ferroptosis inhibitors have the potential to act synergistically to delay or prevent ferroptosis-mediated neurodegenerative mechanisms.

## 5 Conclusion

Control of iron homeostasis is essential for the maintenance of neuronal function and integrity of the CNS. The effects of iron metabolism disruption on oxidative stress, mitochondrial dysfunction, lipid peroxidation, and ultimately ferroptosis all play a role in the initiation and progression of many neurodegenerative and neuroinflammatory diseases. There is mounting evidence that ferroptosis and neuroinflammation are tightly linked processes that can perpetuate each other, wherein iron dysregulations, reactive oxygen species, inflammatory mediators, and lipid peroxidation all drive further neuronal injury.

The intricate molecular crosstalk between ferroptosis and neuroinflammation underscores the importance of iron regulatory mechanisms, antioxidant defense systems, signaling pathways such as Nrf2, NF-κB, SIRT1, COX-2, and iNOS, and their intricate interactions in disease pathogenesis. While each CNS disease shows its own unique pathological features, shared therapeutic targets include iron overload, mitochondrial dysfunction, oxidative stress and immune activation.

In conclusion, the evidence available suggests that therapeutic interventions that target both pathways associated with ferroptosis and neuroinflammation together will offer more neuroprotection than those that target just one of these pathways. The detailed knowledge of the molecular dialogue between these interconnected processes will help in developing mechanism-based therapeutic interventions for CNS disorders.

## 6 Future perspective

Although significant advances have been made in deciphering the link between ferroptosis and neuroinflammation, a number of challenges still exist before ferroptosis-modulating therapies can be successfully applied in the clinic. Future studies should aim to develop a coordinated roadmap with the following areas: (i) identification of reliable and disease-specific biomarkers of ferroptosis and neuroinflammation; (ii) elucidation of the temporal and cell type-specific interactions between neurons, astrocytes, microglia, oligodendrocytes and endothelial cells; (iii) development of therapeutics with improved BBB penetrance and target specificity; (iv) evaluation of combination therapies targeting ferroptosis, neuroinflammation, oxidative stress and mitochondrial dysfunction; and (v) validation of these approaches in well-designed preclinical studies and multicenter clinical trials.

Continued research on systemic factors such as peripheral immune mechanisms, metabolic control, and the gut–brain axis will yield further understanding of disease pathogenesis and treatment. Patient stratification, treatment monitoring, and optimization of individual therapeutic strategies will rely on the integration of molecular biomarkers, advanced neuroimaging, cerebrospinal fluid analysis, and precision medicine approaches.

The discovery of new biomarkers, in recent years, also supports the idea of precision medicine in the management of neurodegenerative diseases. In the future, the clinical translation of interventions targeting ferroptosis may be aided by emerging multimodal approaches that integrate blood- and cerebrospinal fluid-based biomarkers with advanced neuroimaging, multi-omics technologies, and artificial intelligence, which can help in early diagnosis, patient stratification, and therapeutic monitoring (Patwekar et al., 2026a).

In addition, progress in antibody-based nanoplatforms has established potentially effective methods for overcoming barriers posed by the blood–brain barrier for targeted drug delivery and for enhancing molecular imaging and manipulation of neuroinflammatory pathways. These nanotherapeutic strategies are still in a very early stage of development and lay a solid ground for the future clinical practice of precision-based strategies in the treatment of ferroptosis-induced neurodegeneration (Patwekar et al., 2026b).

Taken together, these research priorities offer a practical blueprint for how discoveries of mechanisms will lead to clinically useful therapies. There is significant potential to develop disease-stage-dependent, multi-targeted interventions that are designed to tackle both ferroptosis and neuroinflammation and take into account the cell and disease heterogeneity and metabolic status of patients with CNS disorders.
